# Long-read sequence assembly: a technical evaluation in barley

**DOI:** 10.1093/plcell/koab077

**Published:** 2021-03-12

**Authors:** Martin Mascher, Thomas Wicker, Jerry Jenkins, Christopher Plott, Thomas Lux, Chu Shin Koh, Jennifer Ens, Heidrun Gundlach, Lori B Boston, Zuzana Tulpová, Samuel Holden, Inmaculada Hernández-Pinzón, Uwe Scholz, Klaus F X Mayer, Manuel Spannagl, Curtis J Pozniak, Andrew G Sharpe, Hana Šimková, Matthew J Moscou, Jane Grimwood, Jeremy Schmutz, Nils Stein

**Affiliations:** 1 Leibniz Institute of Plant Genetics and Crop Plant Research (IPK), Gatersleben, Seeland 06466, Germany; 2 German Centre for Integrative Biodiversity Research (iDiv), Halle-Jena-Leipzig, Leipzig 04103, Germany; 3 Department of Plant and Microbial Biology, University of Zürich, Zürich 8008, Switzerland; 4 HudsonAlpha Institute for Biotechnology, Huntsville, AL 35806; 5 PGSB–Plant Genome and Systems Biology, Helmholtz Center Munich–German Research Center for Environmental Health, Neuherberg 85764, Germany; 6 Global Institute for Food Security, University of Saskatchewan, Saskatoon SK S7N 4L8, Canada; 7 Department of Plant Sciences, Crop Development Centre, University of Saskatchewan, Saskatoon SK S7N 5A8, Canada; 8 Institute of Experimental Botany of the Czech Academy of Sciences, Centre of the Region Haná for Biotechnological and Agricultural Research, Olomouc 78371, Czech Republic; 9 The Sainsbury Laboratory, University of East Anglia, Norwich NR4 7UH, UK; 10 Center for Integrated Breeding Research (CiBreed), Georg-August-University Göttingen, Göttingen 37073, Germany

## Abstract

Sequence assembly of large and repeat-rich plant genomes has been challenging, requiring substantial computational resources and often several complementary sequence assembly and genome mapping approaches. The recent development of fast and accurate long-read sequencing by circular consensus sequencing (CCS) on the PacBio platform may greatly increase the scope of plant pan-genome projects. Here, we compare current long-read sequencing platforms regarding their ability to rapidly generate contiguous sequence assemblies in pan-genome studies of barley (*Hordeum vulgare*). Most long-read assemblies are clearly superior to the current barley reference sequence based on short-reads. Assemblies derived from accurate long reads excel in most metrics, but the CCS approach was the most cost-effective strategy for assembling tens of barley genomes. A downsampling analysis indicated that 20-fold CCS coverage can yield very good sequence assemblies, while even five-fold CCS data may capture the complete sequence of most genes. We present an updated reference genome assembly for barley with near-complete representation of the repeat-rich intergenic space. Long-read assembly can underpin the construction of accurate and complete sequences of multiple genomes of a species to build pan-genome infrastructures in Triticeae crops and their wild relatives.

## Teaser

A greatly improved reference genome sequence of barley was assembled from accurate long reads.

##  

**Figure:**
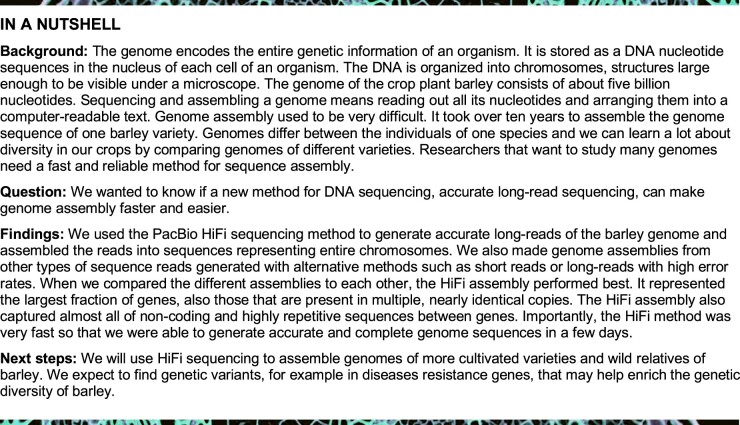


## Introduction

The first plant genome projects, despite focusing on model plants with small genomes, were undertaken by international consortia on the time scale of one decade (The Arabidopsis Genome, 2000; Sasaki and International Rice Genome Sequencing, 2005). In the following two decades, genome sequencing has been a fast-moving field of research propelled by technological advances driving scientific discovery. Very recently, multiple reference assemblies were published for several crop plant species with large genomes, among them tomato (*Solanum lycopersicum*; Alonge et al., 2020), soybean (*Glycine max*; Liu et al., 2020), wheat (*Triticum aestivum*; Walkowiak et al., 2020), and barley (*Hordeum vulgare*; Jayakodi et al., 2020). These studies have highlighted the prospects of the comparative analysis of genome sequence assemblies derived from multiple individuals for resolving long-standing questions in genetic research, such as domestication genes in tomato (Alonge et al., 2020), and the discovery of hitherto inaccessible classes of genetic variants such as large chromosomal inversions (Jayakodi et al., 2020) and introgressions from crop wild relatives (Alonge et al., 2020; Walkowiak et al., 2020).

Pan-genomic studies of large-genome (>1 Gb) plant species have only very recently become possible. Notable examples include the Triticeae crop species wheat, barley, and rye (*Secale cereale*). Their (sub-)genomes have a haploid size between 4 and 7 Gb, with 80%–90% of the sequence derived from transposable elements. Wheat, the most economically important Triticeae crop, is an allohexaploid; the others are diploids. Suppressed recombination within large parts of their genomes makes genetic mapping an ineffective tool for arranging sequence scaffolds along the chromosomes. Owing to these challenges, chromosome-scale whole-genome shotgun (WGS) assemblies of the wheat and barley genomes were only published in the last three years (The International Wheat Genome Sequencing Consortium (IWGSC), 2018; Monat et al., 2019). A crucial technological advance enabling WGS assembly from short-reads in Triticeae crops was the development of an effective protocol for iterative scaffolding of primary contigs using mate-pair libraries, 10X Chromium linked reads and chromosome-conformation capture sequencing (Hi-C) to arrange sequence scaffolds into chromosomal pseudomolecules (Avni et al., 2017; Maccaferri et al., 2019; Monat et al., 2019).

Long-read sequencing has superseded short-read approaches in many plant species (Jung et al., 2019), and has even been deployed on the scale of pan-genome projects (Alonge et al., 2020). Long-read assemblies can capture sequence variation inaccessible to short-read approaches such as regulatory variants residing in the repeat-rich intergenic space or copy-number variants at complex loci. The first long-read assemblies of Triticeae genomes were reported for diploid and hexaploid (Zimin et al., 2017) wheat, but they did not achieve chromosome-level contiguity. The size of primary contigs in long-read assemblies clearly exceeded that of short-read contigs, but evident drawbacks were the immense computational costs (months of wall clock time) and need for further short-read based scaffolding to construct chromosome-scale assemblies. Yuan et al. (2020) have developed a reference-guided approach that is more computationally efficient but has the conceptual disadvantage of masking haplotypes that are diverged from the guide sequence. Thus, first-generation pan-genome projects of wheat and barley had to strike a compromise in their choice of sequencing strategy between representation of intergenic space, scaffold-level contiguity, and size of the pan-genome diversity panel. They chose short-reads, sacrificing completeness of the intergenic space for chromosome-scale contiguity at the scaffold level. Despite its shortcomings, this strategy enabled the discovery of large inversions, translocations, and novel alien introgressions (Jayakodi et al., 2020; Walkowiak et al., 2020).

Selection of a sequencing strategy for future pan-genomic studies in large-genome plant species must consider recent advances in long-read sequencing and assembly algorithms. A development of particular relevance is accurate long-read sequencing on the PacBio platform by circular consensus sequencing (CCS, Wenger et al., 2019). CCS (or Hi-Fi) refers to the repeated read-out of the nucleotide sequences of single DNA fragments of a median length of 20 kb to derive a highly accurate (>99.5%) consensus sequence. The use of these pre-corrected long-reads dramatically reduced the runtime of state-of-art assembly algorithms by two orders of magnitude without any compromises in assembly quality (Nurk et al., 2020; Ruan and Li, 2020). CCS assembly of complex plant genomes has the potential to deliver sequence completeness, higher order contiguity, and computational cost effectiveness without having to compromise between them.

Barley is an excellent model for evaluating sequence assemblies as many datasets to benchmark the accuracy and completeness of genome assemblies are readily available (Stein and Muehlbauer, 2018). A wealth of genome sequencing and mapping resources including a library of full-length cDNAs (Matsumoto et al., 2011), multi-tissue transcript atlas (Mascher et al., 2017), high-density and high-resolution genetic maps (Mascher et al., 2013, 2017), and optical maps (Mascher et al., 2017) have been compiled in the past decade. Most of these datasets have been generated for a single cultivar: Morex, a six-rowed US malting barley commonly grown until the 1980s. Its current reference sequence assembly (Morex V2) was constructed from short-reads using the TRITEX method (Monat et al., 2019). Long-read assemblies have been reported for other barley genotypes (Dai et al., 2018; Zeng et al., 2020), but no systematic comparison between long-read and short-read approaches has been conducted yet in barley or other large-genome plant species.

Here, we assess the performance of multiple long-read sequencing approaches (PacBio continuous long-reads, PacBio CCS, and Oxford Nanopore) in barley, focusing on whether long-read approaches can underpin the construction of chromosome-scale sequence assemblies in barley.

## Results

### Descriptions of raw data, assemblies, and benchmarks

We compared the performance of current long-read sequencing platforms at constructing highly contiguous sequence assemblies of the barley genome. Towards this aim, we analyzed three long-read datasets for barley cv. Morex (Table 1 and Supplemental Table S1): (1) PacBio continuous long-reads (acronym: CLR), (2) PacBio CCS reads (acronym: CCS), and (3) Oxford Nanopore reads (acronym: ONT). The ONT and CCS reads were generated in the present study. The CLR data have been reported before by Jayakodi et al. (2020). To polish long-read assemblies and perform hybrid long-read/short-read assemblies, we used published high-coverage lllumina short-read data of cv. Morex (Monat et al., 2019), acronym: (TRITEX).

**Table 1:** Sequence datasets analyzed in the present study

Acronym	Description	Reference
TRITEX	Illumina short-read data from multiple library types (paired-end, mate-pair, 10× Chromium); used for hybrid assemblies	Monat et al. (2019)
PE450	Overlapping 2 × 250 reads with an insert size ∼450 bp; used for polishing of long-read assemblies; subset of TRITEX	Monat et al. (2019)
CLR	PacBio continuous long reads; 121× coverage	Jayakodi et al. (2020)
CCS	PacBio circular consensus reads; 27× coverage	This study
ONT	Oxford Nanopore reads; 85× coverage	This study
Hi-C	Chromosome conformation capture sequencing data; used for pseudomolecule construction	Mascher et al., (2017)

The datasets listed in Table 1 were assembled using eight different assembly algorithms (Table 2). The CLR data were assembled with MECAT (Xiao et al., 2017) and wtdbg2 (Ruan and Li, 2020). Hybrid assemblies of CLR and TRITEX assemblies were generated with Wengan (Genova et al., 2019). Assemblies of CCS reads were done with Hi-Canu (Nurk et al., 2020) and Falcon (Chin et al., 2016). ONT data were assembled with Smartdenovo (https://github.com/ruanjue/smartdenovo). Acronyms of each assembly are listed in Table 2. The CLR_MECAT assembly was reported before by Jayakodi et al. (2020); all other assemblies were generated in the present study.

**Table 2:** Metrics of different sequence assemblies of the genome of barley cv. Morex

Acronym	Input data	Size^a^	Size > 1 Mb^b^	contig N50^c^	scaffold N50^c^	BUSCO^d^	Isoseq^e^	label sites^f^	HC genes^g^
TRITEX	TRITEX	4.65 Gb	4.23 Gb	33 kb	40.2 Mb	96.0%	96.7%	89.2%	98.3%
CLR_MECAT	CLR, PE450	4.14 Gb	3.94 Gb	10.2 Mb		95.3%	95.6%	95.8%	95.2%
CLR_wtdbg2	CLR, PE450	4.07 Gb	3.32 Gb	2.85 Mb		92.9%	93.8%	91.6%	91.2%
Hybrid_Wengan	CLR, PE450 contigs^h^	4.14 Gb	769 Mb	496 kb		94.8%	95.7%	81.0%	94.0%
ONT_smartdenovo	ONT, PE450	4.14 Gb	4.05 Gb	14.2 Mb		97.4%	96.9%	95.6%	91.6%
CCS_Falcon	CCS, PE450	4.19 Gb	4.09 Gb	24.2 Mb		96.5%	97.0%	98.0%	96.9%
CCS_Canu	CCS	4.48 Gb	4.18 Gb	28.7 Mb		96.5%	97.1%	99.0%	97.1%

Note that gene models are defined on the TRITEX assembly and can be affected by structural errors in that assembly. Genes not aligned to TRITEX (1.7%) are due to alignment uncertainty.

^a^ Total assembly size.

^b^ Cumulative size of sequences contained in scaffolds larger than 1 Mb.

^c^ Long-read assemblies are gap-free, hence scaffold and contig N50s are identical.

^d^ Proportion of complete BUSCO gene models (total: 425, viridiplantae_odb10) present in one or more copies.

^e^ Proportion of aligned Isoseq reads (total: 123,875), minimum alignment length: 90%, minimum identity: 97%.

^f^ Proportion of aligned DLE1 label sites of the Bionano map.

^g^ Proportion of aligned Morex V2 HC gene models (total: 32,787), minimum alignment length: 99%, minimum identity: 100%.

^h^ Contigs assembled from PE450 data with Minia3 (Monat et al. 2019).

Assemblies were evaluated according to the following criteria: (1) basic summary statistics (assembly size, N50/N90 at the contig and scaffold level); (2) gene space representation; (3) coverage of the Bionano map as a proxy for accurate representation of the intergenic space; and (4) alignments of reference (Morex V2) gene models to assess base level accuracy. Gene space completeness was assessed with two complementary datasets: (1) the BUSCO set of conserved single-copy loci (Simao et al., 2015, *N* = 425); and (2) PacBio Isoseq reads of cv. Morex (Mascher et al., 2017, *N* = 123,875). We report the number of complete BUSCO loci in Table 2; full BUSCO results are reported in Supplemental Figure S1.

To derive a metric of sequence accuracy and completeness in the repeat-rich intergenic space, we compared sequence assemblies to an optical map. Mascher et al. (2017) previously reported a Bionano optical map assembled from read-outs of molecules labeled by the Nicking, Labeling, Repairing, and Staining approach (NLRS) and imaged on the Bionano Irys platform. We generated a new Bionano optical map on the Saphyr platform using the Direct Label and Stain (DLS) approach. The contiguity of the DLS map greatly exceeded that of the NLRS map (Table 3). The Bionano DLS map was aligned to each assembly and the proportion of aligned label sites was determined.

**Table 3:** Summary statistics of Bionano optical maps of cv. Morex

	DLS (DLE-1)	NLRS (Nt.BspQI)^a^
Number of filtered molecules	2,791,276	774,557
Molecule N50	281 kb	340 kb
Number of contigs	257	2,875
Contig N50	87.6 Mb	2.1 Mb
Assembly length	4,249 Mb	4,289 Mb
Genome coverage	116×	57×

^a^ Reported by Mascher et al. (2017).

To compare base-level sequence accuracy between assemblies, we aligned high-confidence (HC) models of the current Morex V2 annotation to each assembly and required alignment threshold (99% alignment/100% sequence identity). Our choice of gene model alignments as a benchmark of base-level accuracy was motivated by the observation that differences in gene space representation as approximated by alignment rates of full-length cDNAs and Isoseq reads (at 90% coverage and 97% identity) were minor, indicating that all assemblies captured the vast majority of protein-coding genes. Stringent thresholds on sequence identity of alignments, however, are sensitive to single-base substitutions and indels that are common in long-read assemblies (Watson and Warr, 2019). Using a similar argument, the alignment of Bionano contigs can also serve as a proxy for sequence accuracy in the non-coding space: unaligned label sites can be due to the sequence gaps common in scaffolded short-read assemblies. As contigs of long-read assemblies are free of gaps, missed label sites are best explained by sequence errors in label sites. These metrics were computed for all assemblies and results are discussed in the following.

### Accurate long-reads perform best

Our benchmark metrics are summarized in Table 2. Overall, all long-read assemblies yielded satisfactory results with good contiguity. Contig N50 values ranged from 496 kb (Hybrid_Wengan) to 28.7 Mb (CCS_Canu). Gene space representation was similar to the current barley reference genome based on the TRITEX short-read assembly (93.8%–97.1% aligned Isoseq reads) The CCS assemblies are clearly superior to other long-read assemblies: their N50 values were approximately twice that of the best assemblies from uncorrected long reads, CLR_MECAT and ONT_smartdenovo. Gene space completeness and coverage of the Bionano map were one to three percentage points above CLR_MECAT in both CCS assemblies, with Canu_CCS yielding slightly better results than Falcon_CCS.

The best assemblies of uncorrected long-reads (ONT_Smartdenovo and CLR_MECAT) are of similar contiguity and gene space representation. CLR_wtdbg2 performed worse, with fewer aligned transcripts and lower contiguity. Hybrid assembly using both CLR and Illumina short-reads showed no clear advantages over CLR-only assemblies. The Hybrid_Wengan assembly used CLR reads to scaffold a set of contigs constructed from a single paired-library, but achieved only below-average contiguity and genome representation.

The CCS_Canu assembly did not include a polishing step with Illumina short-reads, while CLR_MECAT, CLR_wtdbg2, and CCS_Falcon used PE450 reads of Mascher et al. (2017) to correct single-base errors and short insertion/deletions. Nevertheless, our proxies of base level accuracy, stringent alignment of gene models and alignment of Bionano contigs, were best for CCS_Canu. Error correction of the CCS_Canu assembly with the PE450 reads using the partial-order alignment (POA) method implemented in wtdbg2 (Ruan and Li, 2020) did not improve the proportion of perfect alignments of HC gene models, but rather reduced it by 0.02 percentage points. This observation is consistent with reports on human genome assembly that indicated that polishing of CCS assemblies with Illumina data may not be required (Garg et al., 2020) or even be harmful due to misalignment of short reads (Nurk et al., 2020).

Taken together, long-read assembly surpasses short-read or hybrid approaches. The theoretical expectations of greatly improved assemblies from accurate long reads have been borne out. Primary contig assemblies generated from CCS data attain a level of contiguity previously achievable only by a complex process of iterative scaffolding (Monat et al., 2019), and can be expected to be arranged easily into chromosomal pseudomolecule using Hi-C and/or genetic maps.

### In-depth analysis of resistance loci highlights the importance of long reads

Our comparisons at the whole-genome level revealed only minor differences in gene space completeness between the long-read assemblies. Therefore, we performed an in-depth analysis of selected loci to examine specific differences between assemblies. Resistance gene (*R* gene) loci are challenging to assemble as they often harbor clusters of highly similar members of a few gene families such as nucleotide-binding site leucine rich repeat (NBS-LRR) genes or receptor-like kinases. The structure of *R* gene loci can be further complicated by long (>10 kb) segmental and tandem duplications (Meyers et al., 2005). For these reasons, they are an excellent benchmark for the ability of sequencing approaches and assembly algorithms to resolve complex loci.

Much effort has been spent on isolating resistance gene loci in barley (Schweizer and Stein, 2011), usually proceeding by genetic fine-mapping followed by physical mapping and sequencing of bacterial artificial chromosomes (BACs). The local sequence assemblies generated in these projects were often manually curated and thus serve as a gold standard for evaluating the quality of WGS assemblies. We compiled a list of cloned or fine-mapped *R* gene loci in the barley genome (Table 4), retrieved BAC sequences covering each locus from public archives. In addition, individual BACs assigned to physical contigs (Ariyadasa et al., 2014) spanning three *R* gene loci (*rps2*, *Rps6*, and *Rps8*) were sequenced on the PacBio RS II platform (Supplemental Table S2).

**Table 4:** Relative performance of different assemblies in resolving resistance gene loci

Assembly	*Mla*	*Rpg1*	*rpg4/Rpg5*	*rps2*	*Rps6*	*Rps8*	Rank sum	Total rank
ONT_smartdenovo	2	1	1	3	2	2	11	1
CSS_Canu	1	3	2	1	1	4	12	2
CCS_Falcon	5	2	2	2	3	3	17	3
CLR_MECAT	3	4	4	3	5	1	20	4
CLR_wtdbg2	4	6	5	5	4	5	29	5
TRITEX	6	5	6	6	5	6	34	6
Hybrid_Wengan	7	6	7	7	7	6	40	7

**Figure 1:**
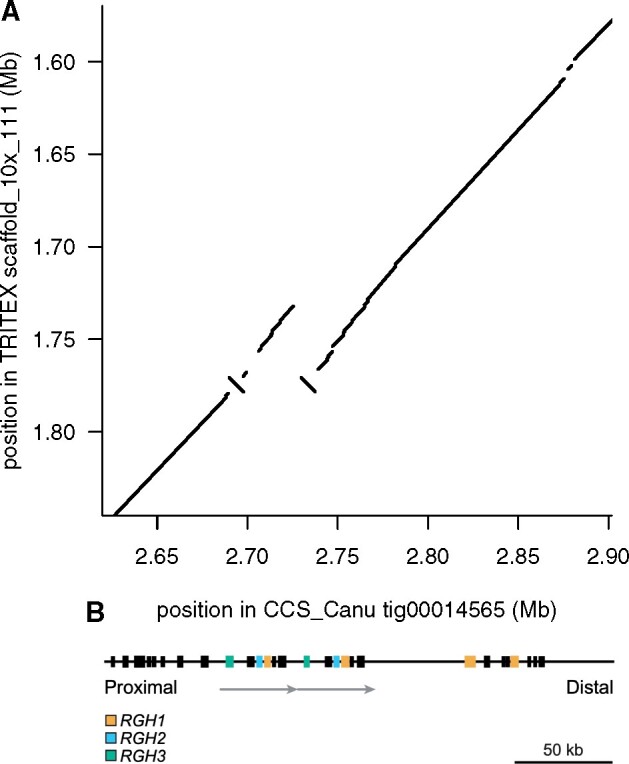
Structural complexity at the *R* gene locus *Mla*. A, A dotplot of the TRITEX (short-read) scaffold versus CCS_Canu (long-read) contig encompassing the *Mla* locus. The region is intact and correct in CCS_Canu, but collapsed in the TRITEX assembly (repeated parallel diagonal lines) and with a small inversion (inverted diagonal line). B, Physical interval of the *Mla* locus from the reference barley accession Morex that contains three gene families *RGH1* (orange), *RGH2* (blue), and *RGH3* (green) encoding nucleotide-binding, leucine-rich repeat proteins. Gray arrows define 39.7 kb tandem duplication. The duplicate regions are 99.9% identical, with only 13 SNPs and 11 InDels difference between the duplicated segments.

BAC sequences were aligned to our whole-genome sequence assemblies and alignments were annotated manually (Figure 1). Assemblies were scored according to alignment completeness and accuracy (Table 4 and Supplemental Table S3). The relative performance of the assemblies was overall in good agreement with the global metrics described above. CCS assemblies were better than CLR assemblies, with Hybrid_Wengan and CLR_wtdbg2 performing worst among long-read assemblies. While differentiated by a small margin in rank due to contig length, the ONT_smartdenovo and CCS_Canu assemblies both assembled all *R* gene loci correctly. In three of the six analyzed loci (*Mla*, *rps2*, *Rps6*), the CCS_Canu contigs produced the best scoring. ONT_smartdenovo was among the top-three assemblies for all six loci and resolved the *Rpg1* locus best. Both CCS assemblies performed worse than ONT_smartdenovo for the *Rps8* locus, where the most accurate sequence was produced by CLR_MECAT.

The most challenging genomic region for assembly was the *Mla* locus, which in Morex contains a 39.7-kb tandem duplication. These duplications are 99.9% identical, with only 13 single-nucleotide polymorphisms (SNPs) and 11 InDels difference between the duplicated segments (Wei et al., 2002). Correct assemblies for the *Mla* locus were observed for CCS_Canu, ONT_smartdenovo, and CLR_MECAT. These findings, while limited to a small number of examples due to the need for manual inspection and available high-quality data, indicate that longer reads from the Oxford Nanopore platform or PacBio continuous long reads can be superior or complementary to accurate CCS reads when genome assemblies are used as a tool to resolve complex loci, particularly those containing tandem duplications larger than CCS read length.

### MorexV3: an improved barley reference sequence

The contiguity and gene space representation of long-read contigs are on a par with the short-read scaffolds that underlie the current barley reference sequence (Morex V2). As long-read contigs resolve repetitive sequence better, an update of the Morex reference assembly is due. As the CCS_Canu assembly outperformed other assemblies in almost all regards (Table 2), it was chosen as the primary contig assembly to construct chromosomal pseudomolecules. Construction of pseudomolecules proceeded in three steps: (1) scaffolding the CCS_Canu assembly with Bionano contigs; (2) removal of small redundant sequences; (3) filling gaps in scaffolds with ONT_smartdenovo contigs; and (4) ordering and orienting scaffolds into chromosomal pseudomolecules. Scaffolding with Bionano contigs resulted in 7,600 scaffolds with an N50 of 105.7 Mb. The CCS_Canu assembly was larger than the other long-read assemblies (Table 2). The additional sequence was contained in small contigs, presumably representing redundant or poorly resolved sequences of repetitive regions such as ribosomal or centromeric repeat arrays.

**Figure 2:**
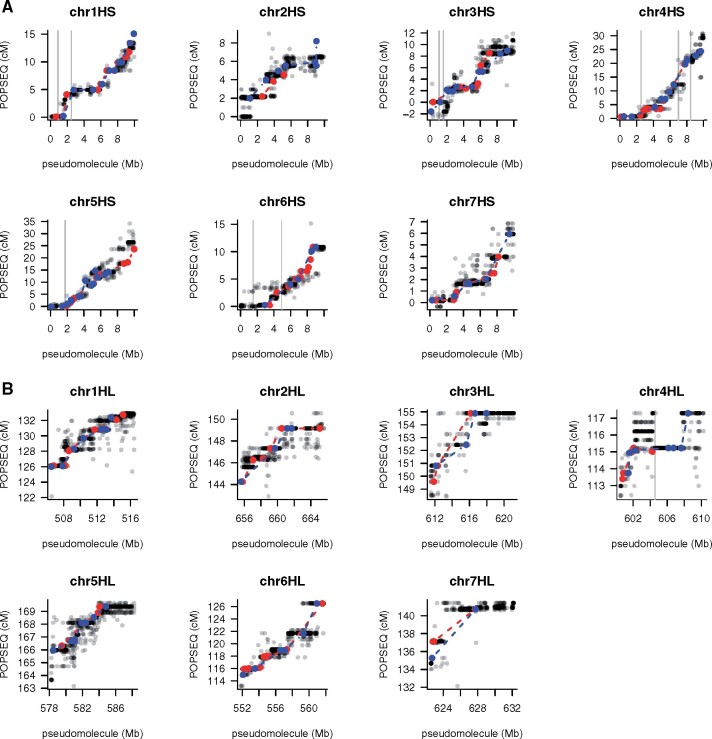
Alignments between Hi-C-based pseudomolecules and genetic maps. Panel A shows POPSEQ markers in the Morex x Barke and Oregon Wolfe Barley (OWB) maps (Mascher et al., 2013). Framework markers of the Morex × Barke and OWB maps are shown in red and blue, respectively. Markers integrated to the consensus POPSEQ markers are shown as gray dots. Panel B shows GBS markers mapped in Morex × Barke recombinant inbred lines (Mascher et al., 2017). Gray lines indicate scaffold boundaries.

Removing small sequence contigs not assigned to chromosomes and/or identified as redundant sequence (“bubbles”) by HiCanu reduced the total assembly size from 4.5 to 4.2 Gb and increased the nominal scaffold N50 to 118.9 Mb (Table 5). Gap filling using TGS Gapcloser (Xu et al., 2019) reduced the gap sequence from 3.37 to 1.32 Mb, more than doubling the contig N50 (Table 5). Published Hi-C data of Morex (Mascher et al., 2017) were used to order and orient scaffolds along the chromosomes using TRITEX. The high contiguity of the sequence scaffolds and availability of high-density genetic maps of the barley genome made it possible to confirm and, if necessary, correct the order and orientations of sequence scaffolds in the high-recombining distal regions (Figure 2).

**Table 5:** Assembly statistics after scaffolding and gap-filling

	Before gap-filling	After gap-filling
Assembly size	4.2 Gb	
Number of scaffolds	386	
Number of contigs^a^	588	439
Scaffold N50	118.9 Mb	
Scaffold N90	21.9 Mb	
Contig N50	31.9 Mb	69.6 Mb
Contig N90	7.2 Mb	19.3 Mb
Gap size	3.37 Mb	1.32 Mb

^a^ Contiguous gap-free stretches within scaffolds.

The terminal 10 Mb, corresponding to 3–20 cM in genetic length, of eight of the 14 chromosome arms (1HL, 2HS, 2HL, 3HL, 5HL, 6HL, 7HS, and 7HL) were spanned by single sequence scaffolds. In addition, mapped markers were fully informative to anchor and orient terminal scaffolds on an additional three chromosome arms (4HS, 4HL, and 5HS). In the remaining chromosome arms (1HS, 3HS, and 6HS), the order of terminal scaffolds was validated, but the genetic maps were not informative about the orientations of some scaffolds due to low genetic resolution or no marker coverage.

**Figure 3:**
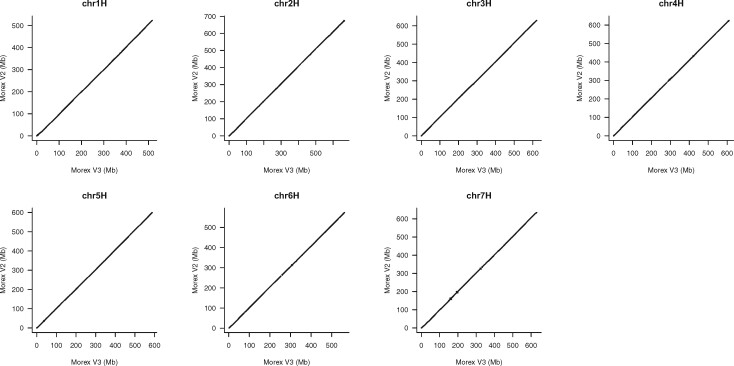
Alignments of MorexV3 and MorexV2 pseudomolecules.

**Figure 4:**
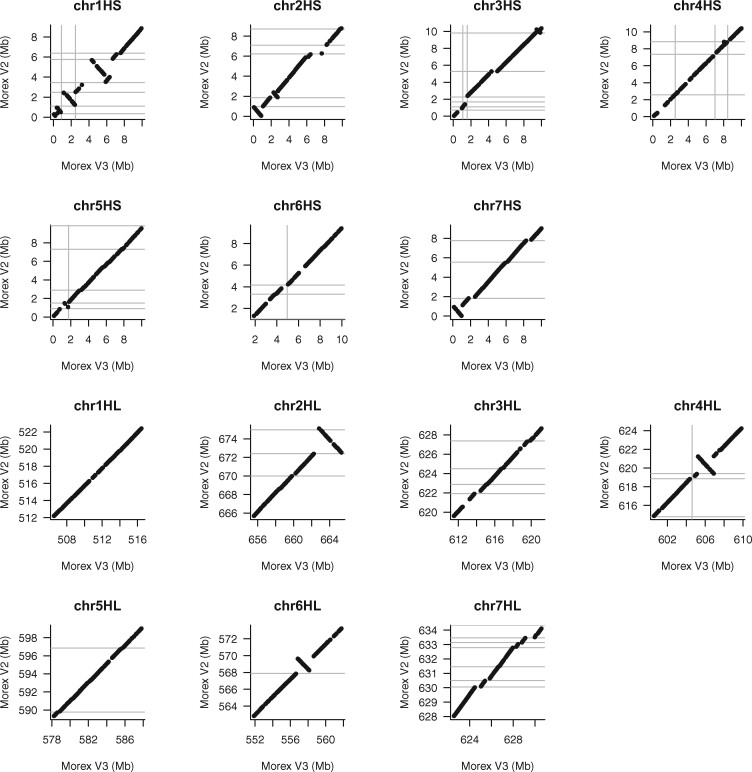
Alignments of Morex V3 and V2 pseudomolecules in the terminal 10 Mb of each chromosome arm. Gray lines indicate scaffold boundaries.

The final pseudomolecules were named MorexV3. Alignments to the MorexV2 assembly showed excellent chromosome-scale collinearity (Figure 3). This is not unexpected because MorexV1 and MorexV2 were also highly collinear; large-scale inconsistencies traced back to a small number of misassemblies in the BAC-based superscaffolds (Monat et al., 2019). The order and orientation of distal sequence in MorexV3, validated by genetic and optical maps (Figure 2), was greatly improved compared with V2 (Figure 4), indicating that the single-step assembly of highly contiguous sequences is superior to iterative scaffolding.

We annotated the MorexV3 pseudomolecules using the same transcriptomic resources as used for MorexV2 (Monat et al., 2019), but with an improved version of the PGSB annotation pipeline, which is also able to call isoforms and UTRs. A total of 81,687 genes with 83,990 transcripts were identified. Of these, 35,827 were classified as HC genes. Among all gene models, 98.6% of BUSCO models were retrieved (Supplemental Figure S2). Detailed annotation statistics can be found in Supplemental Table S4. We analyzed sequence gaps in the intergenic regions surrounding genes, which revealed that 91% of V3 gene models had no ambiguous bases (“Ns”) in their 100 kb flanking sequence compared to only 0.7% in MorexV2 (Supplemental Figure S3). The coding sequences of 35,260 (98.4%) Morex V3 HC gene models had near-complete alignments (≥95% alignment coverage, ≥99% identity) to the V2 pseudomolecules, confirming that gene space presentation is good also in short-read assemblies (Monat et al., 2019).

The final pseudomolecules are composed of between 9 (1H) and 26 (4H) sequence scaffolds arranged and oriented by Hi-C. Gapless assemblies of the centromere of several maize (*Zea mays*) chromosomes have been reported recently (Liu et al., 2020). In contrast to maize, where a sequence contig spanned a centromere, we found neither a Bionano nor a sequence contig spanning any of the barley centromeres, indicating that the size or repeat composition of barley centromeres requires further increases in read lengths to obtain gapless end-to-end assemblies of entire chromosomes. Similar results were obtained for the ribosomal DNA (rDNA) loci on chromosomes 5H and 6H, which contain long stretches, possibly tens of megabases, of tandemly arrayed rDNA units, each ∼9 kb in size. Future work incorporating ultra-long reads might resolve even highly repetitive regions such as centromeres and ribosomal DNA loci (Miga et al., 2020).

### Near-complete assembly of the intergenic space

Our analysis of alignments of DLE-1 label sites (Table 2) gave indications that the intergenic space is better represented in long-read assemblies. Our previous analysis of TEs in the two prior versions of the Morex pseudomolecules (Morex V1 and V2) had revealed pronounced differences in the representation of retrotransposons (Monat et al., 2019) between both assemblies. MorexV1 contained more younger, highly similar copies, presumably due to the hierarchical BAC-based assembly approach that avoided confounding copies at unlinked loci. The WGS-based MorexV2 contained overall more complete elements, but many elements had gaps in their sequence. As the average size of CCS reads is ∼20 kb, the majority of complete BARE1 elements and their flanking sequence can be spanned by single reads. Hence, long-read WGS assembly is likely able to detect even very recent insertions, which are almost absent from the short-read WGS assembly MorexV2.

To better understand these differences between long- and short-read assemblies, we performed two complementary analyses: a global analysis of full-length retrotransposons and an in-depth analysis of the BARE1 family, the most abundant TE family in the barley genome (Manninen and Schulman, 1993; Wicker et al., 2018). We focused on the different pseudomolecule version: Morex V1 (hierarchical short-read assembly of BACs); Morex V2 (WGS short-read); and Morex V3 (whole-genome long-read).

**Figure 5:**
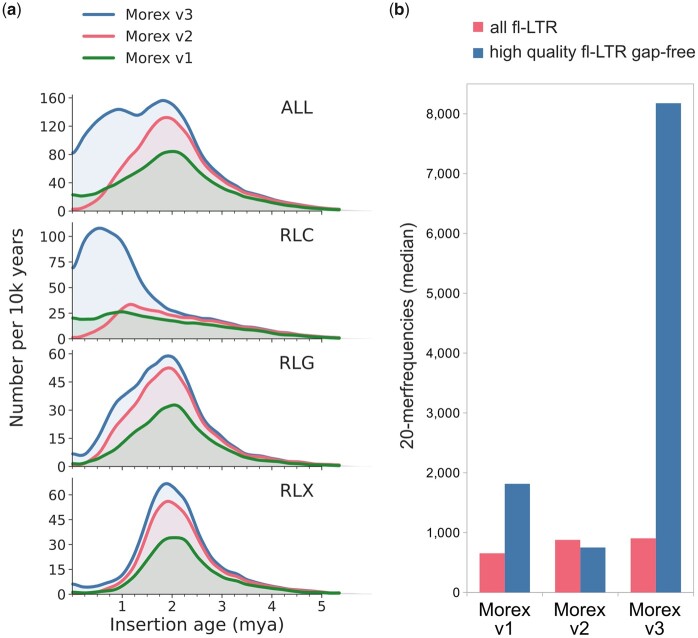
Full-length LTR-retrotransposon (fl-LTR) characteristics of the three Morex chromosome-level assembly versions. A, Fl-LTR insertion age distribution for all high-quality gap-free fl-LTR copies and superfamily subsets (RLC: Copia and RLG Gypsy superfamily, RLX unassigned. B, Overall repetitivity of fl-LTR copies in terms of 20-mer frequencies.

The global analysis revealed strikingly different insertion age distribution inferred from the three pseudomolecule versions (Figure 5, A). The Morex V1 and V2 assemblies contain fewer number of copies as well as different age distributions, especially within the younger RLC superfamily which features highly similar to identical long terminal repeats (LTRs). Morex V3 captures a much higher amount of highly repetitive sequence, which could not be resolved in the short-read assemblies (Figure 5, B). This shortcoming of the two short-read assemblies can be explained by the difficulty to resolve identical repeat structures. Either they are collapsed, leading to lower numbers of intact fl-LTR copies; or they contain sequence gaps leading to a lack of younger copies in the high-quality gap-free fraction. By contrast, the long-read assembly Morex V3 is almost gap-free, and contains a much higher number of younger high-quality fl-LTR copies (Figure 5).

**Figure 6:**
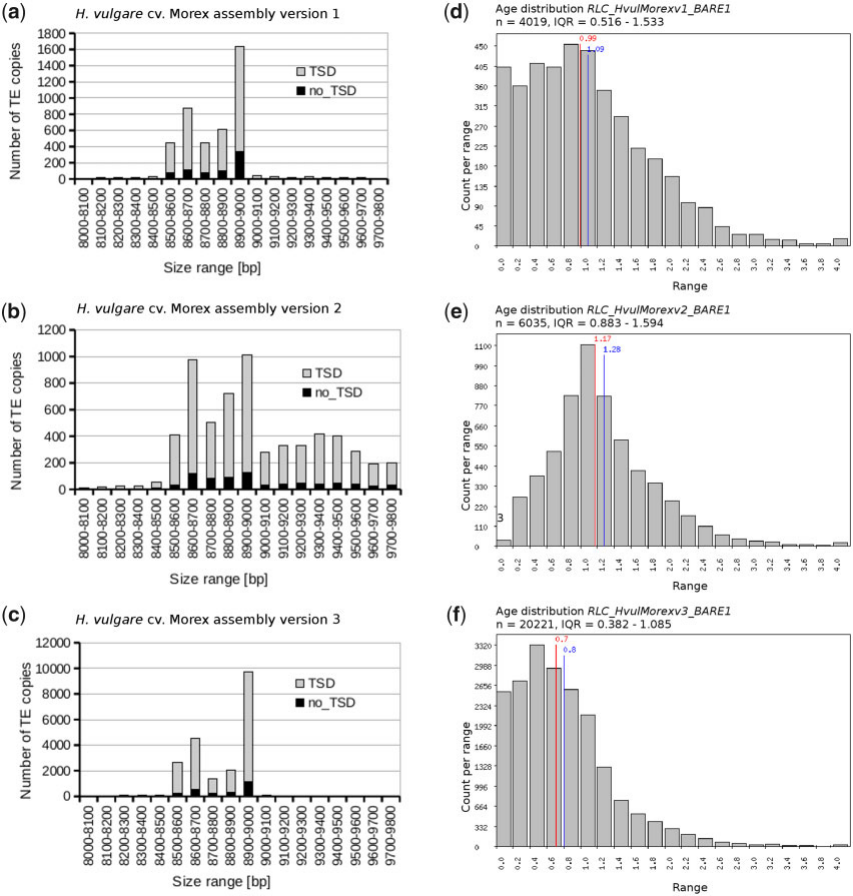
Sizes and insertion age distributions of full-length BARE1 retrotransposons extracted from different Morex assembly versions (V1–V3). Panels A–C show size distributions of the extracted full-length retrotransposons. Those extracted from V2 tend to be much longer due to extended stretches of unfilled gaps represented by N characters. Panels D–F show insertion age distributions of the extracted full-length retrotransposons. Retrotransposons from V1 and V2 are on average older. In V2, very young retrotransposons are almost absent. They could not be identified with our pipeline since LTRs of young elements tend to have sequence gaps.

To analyze the representation of BARE1 elements, we refined the analysis of Monat et al. (2019) and included Morex V3 (Table 6 and Figure 6). Consistent with the global analysis, Morex V3 captured more full-length BARE1 copies than V1 and V2. The size range of the candidate retrotransposons was very similar in V1 and V3, with most copies being between 8.6 and 9 kb long. Furthermore, the distribution had two clear peaks, representing the two main subfamilies of autonomous and non-autonomous elements (Figure 6, A and C). By contrast, candidate retrotransposons in V2 had a much wider size distribution due to more frequent gaps, whose sizes were overestimated (Figure 6, B andTable 1). Insertion age distribution was narrowest in V3, with an average of 700,000 years (Figure 6).

**Table 6:** Numbers of full-length BARE1 retrotransposons and solo-LTRs in the three versions of the Morex pseudomolecules

	Morex V1	Morex V2	Morex V3
Candidates	4,277	6,193	20,944
Excluded^a^	259	139	686
Full-length copies	4,018	6,054	20,258
TSD	3,313 (82%)	5,303 (87%)	17,820 (88%)
Fraction of gaps	0.03%	1.72%	0%
Solo LTRs	3,473	3,742	6,216

^a^ All elements shorter than 8.5 kb were excluded. The maximum allowed length was 9 kb in V1 and V3, and 9.8 kb in V2.

Most RLC_BARE1 retrotransposons are flanked by a 5-bp target site duplication (TSD), which is generated upon insertion of the element (Suoniemi et al., 1997). The presence of a TSD can therefore be used as an indication that correct pairs of LTRs were assembled. Overall, the proportion of LTR retrotransposons flanked by a TSD was similar in all pseudomolecule versions (82%–88%), but highest in V3. This indicates that inter-element mis-assemblies (i.e. unlinked genome regions wrongly connected across a near-identical LTR sequence) occur at similar levels in all versions. Note also that not all mismatches in TSD are due to mis-assemblies: it was estimated that at least 5% of newly inserted LTR retrotransposons do not have perfect TSD (Wicker et al., 2016). An average insertion age of 700,000 years (Figure 6, F) implies that approximately another 5% of TSD have accumulated mutations, in close agreement with the 88% of retrotransposon copies with TSDs in V3.

**Figure 7:**
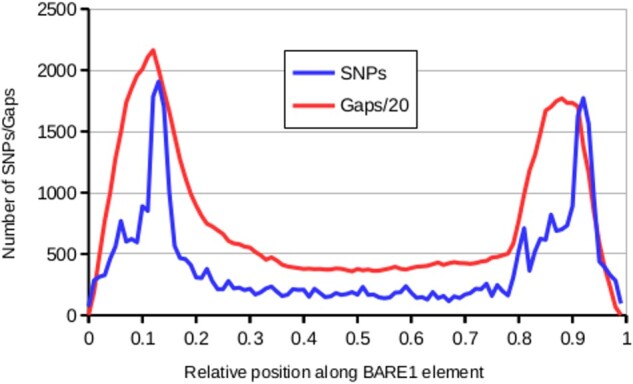
Distribution of sequence gaps and sequence differences in BARE1 elements between Morex V1 and V3. The graph is a compilation of results from sequence alignments of 3,305 v1 and v3 full-length BARE1 retrotransposons. As individual retrotransposon copies can differ in length, the length was normalized to 1,000 bins. The plot shows numbers of SNPs and numbers of N’s in 10-bin windows. The LTRs correspond to approximately the first and last 20% of the retrotransposon. These regions are highly enriched in SNPs and sequence gaps because of the inability of short-read assemblies to resolve highly similar regions longer than a few hundred base pair.

LTRs are problematic for sequence assemblies because the two LTRs of a full-length retrotransposon often are nearly identical. Thus, sequence gaps in LTRs may have prevented the identification of full-length LTR retrotransposons in V1 and V2 assemblies. We searched for homologs of the 20,258 full-length BARE1 elements identified in V3 by retrieving the highly specific sequence junctions between the termini of each element and its flanking sequences in V1. We extracted 3,305 cases where both termini were found in the same orientation and the expected distance from each other. Alignments of matching termini revealed that LTRs are highly enriched in sequence gaps as well as nucleotide differences between V1 and V3 assemblies (Figure 7). These are likely consequences of scaffolding and error-prone gap-filling with short reads in V1. Solo-LTRs are the result of intra-element recombination that eliminates one LTR and the internal domain. These are less problematic to assemble since they do not come in pairs. Indeed, the numbers of identified solo-LTRs differed less strongly between assemblies (Table 6). Nevertheless, V3 contains nearly twice as many solo-LTRs than either V1 or V2.

In summary, our analysis supports a near-complete representation of the intergenic space in the MorexV3 long-read assembly. This represents a substantial improvement over previous assembly versions, whose shortcomings had led to underestimated numbers of full-length copies and biased age estimates.

### Exploring the parameter space of CCS assembly

Long-read assembly in wheat had been reported to be computationally intensive, with run-times on the order of months to complete primary contig assembly (Zimin et al., 2017a, 2017b). In the present study, assemblies using uncorrected long-reads (CLR and ONT) were completed on the time scale of weeks and months. As computations were conducted at four different sites using different infrastructures, exact comparisons between different assemblies are not possible. A striking observation, however, was the extremely low computational cost for CCS assembly. The CCS_Canu assembly underlying the Morex V2 was completed on a single machine within six days using 30 CPU cores and <500 Gb of RAM, in accordance with very low computational costs reported by Nurk et al. (2020). If the initial read trimming step is omitted, as in the current release of Hi-Canu (v2.0, Nurk et al., 2020), compute time can be further reduced to three days on a single server with 72 cores.

These short run times enabled a downsampling analysis. Subsets of the CCS data were assembled with Hi-Canu to determine the impact of input read coverage on assembly metrics. We sequenced two CCS libraries with average fragment sizes of 19 and 22 kb on five SMRT cells with a total yield of 132.7 Gb, amounting to ∼26.5× coverage (assuming a genome size of 5 Gb). We downsampled this dataset by omitting reads from one or multiple SMRT cells. The resultant assemblies were evaluated for assembly size, contiguity (N50), accurate gene space representation (proportion of stringently aligned Morex V2 HC gene), and coverage of the Bionano map as described above (Table 7).

**Table 7:** Metrics of Hi-Canu assemblies of down-sampled CCS data

ID	19k^a^	22k^a^	Reads (Gb)	Coverage	N50 (Mb)	N90 (Mb)	Size (Gb)^b^	Size 1 Mb (Gb)^c^	HC genes^d^	Label sites^e^
3_2_trim^f^	3	2	132.7	26.5	28.7	3.6	4.48	4.17	97.1%	99.0%
3_2^f^	3	2	132.7	26.5	31.1	3.9	4.50	4.17	97.1%	99.0%
3_1	3	1	109.3	21.9	25.8	4.4	4.42	4.16	97.0%	98.7%
2_2	2	2	99.2	19.8	21.6	3.8	4.39	4.15	96.9%	98.5%
3_0	3	0	89.8	18.0	19.5	3.7	4.35	4.15	96.8%	98.5%
2_1	2	1	79.9	16.0	13.1	2.9	4.33	4.13	96.2%	98.3%
1_2	1	2	69.8	14.0	8.0	1.9	4.30	4.05	95.3%	98.2%
2_0	2	0	63.0	12.6	5.8	1.4	4.28	4.00	94.8%	98.1%
1_1	1	1	52.8	10.6	2.5	0.6	4.26	3.50	92.0%	97.5%
1_0	1	0	33.6	6.7	0.4	0.1	4.18	0.48	80.5%	88.2%

^a^ Two libraries with average insert sizes of 19 and 22 kb, respectively, were prepared. The 19 k library was sequenced on three SMRT cells, the 22 k library on four. The columns report the number of SMRT cells whose reads were included in the assemblies.

^b^ Total assembly size.

^c^ Cumulative size of sequences contained in scaffolds larger than 1 Mb.

^d^ Proportion of aligned Morex V2 HC gene models, minimum alignment length: 99%, minimum identity: 100%.

^e^ Proportion of aligned DLE1 label sites of the Bionano map.

^f^ 3_2_trim is the CCS_Canu assembly used for constructing the Morex V3 pseudomolecules. In 3_2, the trimming step was omitted in HiCanu.

Omitting the trimming step (Table 7: 3_2_trim vs. 3_2) yielded an assembly with nearly equal metrics. Assemblies from four SMRT cells (∼20× coverage, Table 7: 3_1, 2_2) resulted in assemblies with an N50 above 20 Mb and similar gene space completeness and coverage of the optical map as in the complete dataset. As coverage decreased, all assembly metrics worsened. However, even with only one SMRT cell (∼4–5× coverage), 80.5% of HC genes, and 88.2% of Bionano label sites were aligned. Shallow-coverage CCS could be suitable for applications where the sequences of many, but not all, genes are needed. It could, for example, replace targeted enrichment strategies (Bernhardt et al., 2020) to obtain complete gene sequences for phylogenetic studies. Sequencing CCS libraries to 20-fold or higher genome coverage seems the most effective approach towards chromosome-scale assembly as our analysis of distal regions (Figure 4) showed that improvements of primary assembly contiguity translate into pseudomolecules that are concordant with high-resolution genetic maps and correct errors of prior assembly versions. Finally, we note that, although the Bionano map was an excellent tool to validate the structural integrity of our assembly and increase its contiguity, it is not required for chromosome-level scaffolding: the CCS_Canu assembly was also arranged into accurate pseudomolecules using only Hi-C data (Supplemental Figure S4).

## Discussion

Our comparison of assembly methods can be summarized as follows: The best long-read assemblies were clearly superior to the current barley reference sequence based on short-reads. The choice of assembly algorithm has a strong influence on assembly results. Hybrid approaches, where short-read assembly are scaffolded with long reads, are not worthwhile as the added cost and effort for obtaining and utilizing short-reads does not translate into improvements of assembly quality. Assemblies made from accurate long reads outperform those made from uncorrected reads in most metrics (Table 2), but longer uncorrected reads may provide crucial information to resolve complex loci (Table 4). Importantly, the long contigs of the Morex V3 assembly, uninterrupted by interspersed gap sequence, afford a near-complete representation of the intergenic space, enabling in-depth studies of TE evolution and regulatory elements.

Consistent with reports in wheat (Zimin et al., 2017; Yuan et al., 2020), our assemblies of long reads with high error rates (CLR, ONT) completed only on the timescale of weeks to months. By contrast, a single assembly of barley CCS data with Hi-Canu finished within one to six days, depending on sequence depth. Rather than compromising quality in favor of runtime, our data support that CCS is currently both the fastest and the best assembly strategy in barley. As the contiguity of a primary CCS assembly exceeds those of the short-read scaffolds of the first-generation barley pan-genome assemblies, we believe that Hi-C scaffolding of CCS assemblies is currently the most promising strategy for obtaining multiple chromosome-scale sequence assemblies in barley. Highly contiguous and accurate assemblies can be obtained from CCS data with 20-fold haploid genome coverage. If pseudomolecule-level assembly is not required, Hi-C data can be omitted.

At the time of writing, the cost of sequencing consumables for the CCS+Hi-C approach is approximately EUR 10,000–15,000 for one barley genome. This four-fold reduction compared with short-read sequencing of multiple libraries will enable greater coverage of the barley diversity space by pseudomolecule-level assemblies. Apart from pan-genomics focused on diversity panels, we are proposing that long-read sequence assemblies should become a tool for “traditional” barley genetics, which has had a strong focus on positional cloning in biparental populations. Contiguous sequence assemblies of parental genotypes support the accurate delimitation of mapping intervals and the identification and validation of candidate genes (Thind et al., 2018).

Mutation breeding has had a profound and long-lasting impact on genetic research in barley (Druka et al., 2011), and mapping-by-sequencing with short-reads has identified causal variants underlying many classical mutant phenotypes (Pankin et al., 2014; Jost et al., 2016). Large structural variants generated by radiation mutagenesis are “blind spots” for Illumina sequencing as small to medium-sized deletions (<10 kb) in the intergenic space or balanced events such as inversions and translocations are difficult or impossible to detect with mapping short-reads to a reference sequence, particularly when the reference is possibly diverged from the mutant’s wild-type background. Long-read assembly of mutants and their wild-type background may advance gene isolation in mutants that have proven recalcitrant to map-based cloning (Babb and Muehlbauer, 2003; Shahinnia et al., 2012).

A pertinent question is whether our findings in barley can be generalized to other plant species. From the viewpoint of genome assembly, bread wheat is not substantially more complex than barley because of the divergence between the three subgenomes. Previous approaches to tackle the barley genome such as chromosomal genomics (Mayer et al., 2011), mapping-by-sequencing (Mascher et al., 2013) and Hi-C scaffolding (Mascher et al., 2017) have yielded satisfactory results when applied to wheat (International Wheat Genome Sequencing Consortium (IWGSC), 2014; Chapman et al., 2015; Avni et al., 2017; The International Wheat Genome Sequencing Consortium (IWGSC), 2018). Hence, we anticipate that, with additional expenses for consumables and computational resources, CCS assembly will also be able to underpin accurate chromosome-scale sequence assembly in wheat and other polyploid Triticeae. The cost-effectiveness and scalability of CCS sequencing makes the construction of reference sequences for all donor species of alien introgressions in bread wheat (Molnár-Láng et al., 2015) an attainable research goal.

A notable drawback of TRITEX and other short-read based methods was their inability to produce phased assemblies that separate homologous sequences of heterozygous genomes. Several species in the genus Hordeum are outcrossing and/or autopolyploid, notably barley’s closest relative *H. bulbosum* (Blattner, 2018). Doubled haploids from diploid cytotypes of that species have been used in genomic studies, but are very difficult to maintain (Wendler et al., 2017). Similarly, inbred lines can be obtained for certain rye (*Secale cereale*) genotypes, but would represent only a tiny fraction of rye’s genetic diversity (Rabanus-Wallace et al., 2019). The only viable option to construct a reference genome and conduct population genomic studies is long-read sequencing. Haplotype-resolved assembly of heterozygous genomes will likely require a higher read coverage than in an inbred (e.g. 20× per haplotype) and will benefit from supporting datasets such as short-read data of the parents (Koren et al., 2018) or the gametes (Campoy et al., 2020) of the sequenced individual. Future research should focus on evaluating accurate long-read sequencing in more diverse plant species, including those with heterozygous genomes. Cost-effective sequence assembly with accurate long-reads can open new horizons for phylogenomics.

## Materials and methods

### Library preparation and sequencing

#### High molecular weight DNA extraction

High molecular weight (HMW) DNA for PacBio CCS and ONT sequencing was isolated from fresh leaf tissue harvested from ∼100 seedlings of barley (*H. vulgare*) cv. Morex following a hybrid protocol that combines nuclei isolation followed by a phenol chloroform large-scale extraction (Dvorak et al., 1988; Avni et al., 2017).

#### Pacbio CCS

Long-read sequencing was performed using CCS mode on a PacBio Sequel II instrument. Libraries were constructed using SMRTbell Template Prep Kit 1.0 followed by tight sizing on a SAGE ELF instrument. Sequencing was performed on five SMRT cells using a 30 h movie time with 2 h pre-extension and sequencing chemistry V2.0. The resulting raw data were processed using the CCS4 algorithm.

#### ONT

Stock HMW DNA was size selected by pulse field electrophoresis on a Blue Pippin instrument (Sage Science) using the high pass protocol to remove fragments <15 kb. Eluate was bead cleaned and quantified by fluorometry (Qubit 2.0) and DNA integrity and size was evaluated with a Tapestation 2200 instrument (Agilent). Library preparation followed the Genomic DNA by Ligation protocol (SQK-LSK-109; Oxford Nanopore Technologies version: GDE_9063_v109_revQ_14Aug2019) optimized for long fragment recovery, with minor modifications. Approximately 20–50 fmol/flow cell of 1D library was targeted for sequencing on a total of 20 sequencing runs (PromethION: FLO-PRO-002 [*n* = 17], GridION FLO-MIN106 R9.4.1 revD [*n* = 3]) following standard default run parameters and high accuracy live-basecalling (Guppy basecaller v.3.2). A total of 507 Gb raw sequence data (40,384 raw reads) were generated with a median read N50 length of 25.5 kb.

### Sequence assembly

#### CLR_MECAT

The CLR_MECAT assembly reported by Jayakodi et al. (2020) and is accessible under EMBL-ENA accession ERS5134609.

#### CLR_wtdbg2

wtdbg2 version 2.5 (20190621) was used. All-against-all of CLR reads longer than base pair (corresponding to 50× coverage) was done with kbm_aln using the parameters -z 15 -c -S 2 -k 0 -p 21 -K 1000.05. Assembly was done with default parameters. Two rounds of error correction with wtpoa-cns were done. In first the round, alignment of the CLR input data were used for correction.

Prior to alignment, PE450 reads were merged with BBMerge (Bushnell et al., 2017) and corrected with BFC (Li, 2015) as described by Monat et al. (2019). Both PE450 and CLR reads were aligned with Minimap2 (Li, 2018). Alignment records were converted to BAM format with SAMtools (Li et al., 2009) and sorted with Novosort (http://www.novocraft.com/products/novosort/).

#### CCS_Canu

CCS reads were assembled with Hi-Canu git commit r9818 using default parameters

#### CCS_Falcon

CCS reads were assembled using FALCON (version 1.8.1, Chin et al., 2016) and the resulting assembly was polished using RACON (version 1.4.10, https://github.com/isovic/racon, Vaser et al., 2017). The CCS assembly-specific commands included pread ovlp_daligner_option (-k24 -h1024 -e.98 -s100 -l2000), and for assembly (length_cutoff_pr = 1000, overlap_filtering_setting = –max-diff 300 –max-cov 400 –min-cov 2 –n-core 32 –min-idt 99.97 –ignore-indels). Two rounds of RACON polishing began by aligning reads using pbmm2 with –preset=CCS to the unpolished assembly. Alignments were then filtered using samtools (-F 1796 -q 20), and the RACON polishing was performed using the parameters “-u –t 64” to produce the initial polished FASTA. A second round of polishing was then performed using the same command set to produce the final polished assembly.

#### ONT_smartdenovo

Smartdenovo (v1.0, https://github.com/ruanjue/smartdenovo) was used to assemble the ONT raw reads with k-mer = 23 and minimum 5 kb read length parameters. The smartdenovo assembly was polished with ONT raw reads and PE450 Illumina short-reads of Monat et al. (2019). The wtpoa-cns module of wtdbg2 (Ruan and Li, 2020) was used for iterative polishing. Onee round of polishing with ONT reads was followed by three rounds of short-read polishing. ONT and corrected PE450 reads were aligned to the iteratively corrected assemblies with Minimap2 (Li, 2018) and alignments processed as described for CLR_wtdbg2.

#### Hybrid_Wengan

PacBio CLR reads were used to scaffold the TRITEX contig assembly reported by Monat et al. (2019) with Wengan v0.1 (Genova et al., 2019). Fastmin-sg was run with the parameter “pacraw -k 20 -w 5 -q 40 -m 150 -r 50000 -I 500, 1000, 2000, 3000, 4000, 5000, 6000, 7000, 8000, 10000, 15000, 20000, 30000”. Liger was run with the parameter “-mlp 10000”.

### Bionano optical map construction

HMW DNA was extracted from two million flow-sorted barley nuclei as described in Šimková et al. (2003). A total of 525 ng DNA were directly labeled at DLE-1 recognition sites (CTTAAG) according to the standard Bionano Prep DLS Protocol and the labeled molecules were analyzed on a Saphyr instrument (Bionano Genomics, San Diego, USA). In total, 1.16 Tbp of single molecule data greater than 150 kbp (N50: 239 kb), corresponding to 232× of the barley genome, were collected from three flow cells of two Saphyr chips. This data were used in de novo assembly by Bionano Solve 3.4.1_09262019, including Pipeline version 10026 and RefAligner version 10020, using “optArguments_nonhaplotype_noES_DLE1_saphyr.xml.” The assembly was built using significance thresholds of *P*-value <1e−10 to generate draft consensus maps, *P*-value < 1e−11 for draft consensus maps extension (five rounds) and *P*-value < 1e−15 for final draft consensus maps merging.

### Assembly evaluation

#### Bionano mapping

The Bionano DLS map was aligned with BionanoSolve version 3.5_12162019 to each assembly. In silico digestion of assemblies was done with fa2cmap_multi_color.pl using the parameter “-e DLE 1.” The characterizeDefault argument set of “optArguments_haplotype_DLE1_saphyr_human.xml” was used for alignment with RefAligner. Alignments were imported into R and processed with scripts hosted in a Bitbucket repository (https://bitbucket.org/tritexassembly/tritexassembly.bitbucket.io/src/master/miscellaneous/bionano_example.R). Only optical contigs longer than 100 kb and alignments with a confidence score >= 20 were considered.

#### Alignment of transcripts

Barley full-length cDNAs (Matsumoto et al., 2011), MorexV2 HC coding sequences (Monat et al., 2019), and Isoseq reads (Mascher et al., 2017) were aligned to the genome sequence assemblies using GMAP (Wu and Watanabe, 2005) version September 12, 2019.

#### BUSCO

Completeness of the Morex genome assemblies was measured with BUSCO (Simao et al., 2015) using the “genome mode” (version 4.06, viridiplantae orthodb10).

### Analysis of resistance gene loci

BAC clones spanning the *R* gene loci *rps2*, *Rps6*, and *Rps8* were identified based on the BAC tiling path of barley (International Barley Genome Sequencing Consortium, 2012; Ariyadasa et al., 2014) and obtained from French National Institute for Agriculture, Food and Environment-Unité de Recherche Génomique Info (https://urgi.versailles.inra.fr/). *Escherichia coli* containing BAC plasmids were grown overnight in LB containing 12.5 μg/mL chloramphenicol. Plasmid DNA was isolated using a Large-Construct Kit (Qiagen, Hilden, Germany) according to the manufacturer’s instructions with no modification. Sequencing of individual BAC clones was performed using individual SMRT cells on a PacBio RS II using C6-P4 chemistry (Earlham Institute, Norwich, UK). BAC clones were assembled using the HGAP 3.0 pipeline and manually curated to circularize and backbone removal of the BAC plasmid. Previously sequenced *R* gene loci were acquired from National Center for Biotechnology Information (NCBI) (http://www.ncbi.nlm.nih.gov/). *R* gene loci were manually assessed for continuity and InDels relative to sequenced BAC contigs and individual assemblies. A rank order of quality was generated based on the following ordered measures: (1) correct assembly of the entire physical contig and (2) the size of the contig in which the locus was identified. The second measure was used to differentiate high quality assemblies. Multiple sequence alignments of BACs and WGS assembly were done with MAFFT (v6.903b, Katoh and Toh, 2008) to find SNPs and indels (Supplemental Table S3).

### Pseudomolecule construction

The CCS_canu contigs were scaffolded with BionanoSolve (https://bionanogenomics.com/support/software-downloads/) version 3.5_12162019 using the “hybridScaffold_DLE1_config.xml” parameter set. Prior to gap filling, the set of scaffolds was filtered using the following criteria: scaffolds not assigned to chromosomes using Hi-C and POPSEQ data as described in the TRITEX pipeline were discarded unless they fulfilled the following three conditions (1) their length was ≥50 kb, (2) they were not reported to be “bubbles” by Canu, and (3) they had 10-folded coverage with CCS reads as report by Canu *OR* carried genes not present in scaffolds assigned to chromosomes. Gap filling was done with TGS-Gapcloser (Xu et al., 2019, https://github.com/BGI-Qingdao/TGS-GapCloser) using ONT_smartdenovo contigs as “reads” for closing gaps. The parameters “–min_match 5000 –min_idy = 0.5 –ne” were used. The script TGS-GapCloser.sh was modified to use the following Minimap2 parameters: “-K 10G -I 10G -f 0.005 -x asm5” for assembly-to-assembly alignment and exclusion of highly repetitive minimizers. Gap sequence in the scaffolds before and after gap filling was determined with seqtk (https://github.com/lh3/seqtk, parameters “cutN -g -n 0”). Gap-filled scaffolds were used as input for pseudomolecule construction using the TRITEX pipeline as described by Monat et al. (2019). Hi-C data of (Mascher et al., 2017) were used for ordering and orienting scaffolds (ENA accession PRJEB14169). POPSEQ markers (Mascher et al., 2013) and GBS loci mapped in the Morex × Barke recombinant inbred lines reported by Mascher et al. (2017) were aligned to preliminary pseudomolecules using Minimap2 (Li, 2018) and the order and orientations of scaffolds in the distal 10 Mb of each chromosome arm were inspected and corrected manually.

### Gene annotation

Structural gene annotation was done using the method previously described by Monat et al. (2019). In summary, the annotation pipeline combines three methods for structural gene annotation in plants: protein homology, expression data based and ab initio prediction. The following sets of evidences were used during homology-based annotation step: Triticeae protein sequences (UniProt; December 5, 2016), and coding sequences of two previously reported barley annotations Morex V2 (Monat et al., 2019), a pan-genome informed annotation of Morex (Jayakodi et al., 2020), and BaRTv1.0 (Rapazote-Flores et al., 2019). Protein sequences were mapped using GenomeThreader (version 1.71, Gremme et al., 2005), whereas nucleotide sequences were mapped using GMAP (version 2018-07-04, Wu and Watanabe, 2005). As evidences derived from expression data, RNA-seq data were first mapped using Hisat2 (version 2.0.4, Kim et al., 2019, parameter –dta) and subsequently assembled into transcripts by Stringtie (version 1.2.3, Pertea et al., 2015, parameters -m 150-t -f 0.3). Additionally, Isoseq and full-length cDNA data was mapped using GMAP. Isoseq and RNA-seq datasets for Morex are described in (Mascher et al., 2017). Full-length cDNA were published by Matsumoto et al. (2011).

All transcripts from Isoseq, RNA-seq, and aligned CDS sequences were combined using Cuffcompare (version 2.2.1, Trapnell et al., 2012), subsequently merged with Stringtie (version 1.2.3, parameters –merge -m150) into a pool of candidate transcripts. Transdecoder (version 3.0.0, https://github.com/TransDecoder/TransDecoder/wiki) was used to find potential open reading frames and to predict protein sequences within the candidate transcript set. Ab initio annotation using Augustus (version 3.3.2, Stanke et al., 2006) was carried out to further improve structural gene annotation. In order to minimize over-prediction, hint files using the above mentioned Isoseq, full-length cDNA, RNA-seq, protein sequences, CDS, and TE predictions were generated. In order to ensure a precise prediction, a specific model for barley was trained according to Hoff and Stanke (2019). All structural gene annotations were joined by EvidenceModeller (Haas et al., 2008) with weights adjusted according to the input source. Different isoforms and UTRs were predicted through two runs of PASA pipeline (Haas et al., 2003) using the Isoseq and full-length cDNA sequences as inputs.

Functional annotation of predicted protein sequences was done using the AHRD pipeline (https://github.com/groupschoof/AHRD).

Finally, gene candidates were classified into HC- or low-confidence (LC) genes. Non-redundant candidate protein sequences were compared against the following three manually curated databases using BLASTp: first, PTREP, a database of hypothetical proteins that contains deduced amino acid sequences in which, in many cases, frameshifts have been removed, which is useful for the identification of divergent TEs having no significant similarity at the DNA level; second, UniPoa, a database comprised of annotated Poaceae proteins; third, UniMag, a database of validated magnoliophyta proteins. UniPoa and UniMag protein sequences were downloaded from Uniprot and further filtered for complete sequences with start and stop codons. Best hits were selected for each predicted protein to each of the three databases. Only hits with an *E*-value below 10e−10 were considered. A HC protein sequence is complete and has a subject and query coverage above the set threshold of 80% in the UniMag database, or no blast hit in UniMag but in UniPoa and not TREP. A LC protein sequence is not complete and has a hit in the UniMag or UniPoa database but not in TREP, or no hit in UniMag and UniPoa and TREP but the protein sequence is complete. The tag REP was assigned for protein sequences not in UniMag and complete but with hits in TREP.

On top of this homology-based classification, further refinements were implemented using functional assignments. Human-readable description lines were scanned for TE, plastid and non-protein coding keywords and tagged accordingly. Any non-tagged LC proteins with an AHRD 3* rating was promoted to HC. Contrarily, HC proteins were demoted, if its AHRD rating is only a one star.

Completeness of the predicted gene space was measured with BUSCO (version 4.06, viridiplantae orthodb10 (Simao et al., 2015). Morex V3 HC gene models were aligned with GMAP to the Morex V2 pseudomolecules to assess their representation in the V2 assembly.

### Annotation of retrotransposons

Still intact full-length LTR retrotransposons were identified and characterized using LTRharvest (Ellinghaus et al., 2008). LTRharvest scans the genome sequences for LTR retrotransposon specific structural hallmarks, like LTRs, RNA cognate primer binding sites and target site duplications. It was run with the following parameter settings: “overlaps best -seed 30 -minlenltr 100 -maxlenltr 2000 -mindistltr 3000 -maxdistltr 25000 -similar 85 -mintsd 4 -maxtsd 20 -motif tgca -motifmis 1 -vic 60 -xdrop 5 -mat 2 -mis -2 -ins -3 -del -3.” Candidates for full-length LTR sequence were subsequently annotated for PfamA domains using hmmer3 (http://hmmer.org/). The inner domain order served as a criterion for the LTR-retrotransposon superfamily classification into either Gypsy (RLG: RT-RH-INT) or Copia (RLC: INT-RT-RH). In the cases of insufficient domain information (undetected RH or INT) the elements were assigned as still undetermined (RLX). The candidate sequences were subjected to a stringent filter for gap-free high quality elements varying between 7,516 and 19.977 per assembly by the following criteria: no gaps (=“N” bases); tandem repeat percent inner ≤30 and LTR ≤35; absence of gene Pfam domains; absence of duplicated TE Pfam domains; strand consistency between Pfam domains and primer binding site. The insertion age of the fl-LTR copies was calculated from the genetic distance between the left and right LTR (emboss distmat, http://emboss.sourceforge.net/, Kimura2-parameter correction) using a random mutation rate of 1.3 × 10^−8^ (SanMiguel et al., 1998). K-mer frequencies were calculated for each base pair in the genome assemblies with tallymer (Kurtz et al., 2008). The overall repetitivity of fl-LTR sequences in terms of 20-mer values was extracted subsequently per element as median frequency over all 20-mer values within the element.

### Analysis of BARE-1 elements

Our pipeline for the isolation of full-length LTR retrotransposons identifies (largely gap-free) LTR sequences that are in the same orientation and at a distance that is expected for the respective TE family. In the case of RLC_BARE1 (Wicker et al., 2007), full-length elements range in size from 8.6 to 9 kb, depending on the retrotransposon subfamily. Candidate retrotransposons were size selected to exclude copies with extensive deletions or insertions. Insertion age of all identified retrotransposons was estimated based on divergence of their LTRs as described by Buchmann et al. (2012). To extract flanking sequences of full-length LTR termini, we extracted the terminal 100 bp of the elements and 100 bp of flanking sequences at both end from Morex V3 and conducted BLAST searches (Altschul et al., 1990) against Morex V1. The corresponding V1 and V3 copies were then aligned with the program Water from the EMBOSS suite (emboss.sourceforge.net, Staden et al., 2003).

### Accession numbers

ENA accession numbers for sequence raw data and genome sequence assemblies are reported in Supplemental Table S1. Genome sequence assemblies and the annotated Morex V3 pseudomolecules are also available for download under a Digital Object Identifier (DOI): http://doi.org/10.5447/ipk/2021/3. The second version of the optical map of Morex is accessible under DOI http://doi.org/10.5447/ipk/2021/2. DOIs were registered in the Plant Genomics and Phenomics Data repository (Arend et al., 2016) using e!DAL (Arend et al., 2014). NCBI BioProject PRJNA664952 contains the raw and assembled BAC sequences for *rps2*, *Rps6*, and *Rps8 R* gene loci.

## Supplemental data

**Supplemental Figure S1.** BUSCO assessment of different sequence assemblies. Supports Figure 1.

**Supplemental Figure S2.** BUSCO assessment of the structural gene annotation of the Morex V3 pseudomolecules. Supports Figure 3.

**Supplemental Figure S3.** Gap-free sequence in upstream and downstream regions of genes in Morex V2 and Morex V3. Supports Figure 3.

**Supplemental Figure S4.** Hi-C contact matrices for pseudomolecules constructed from the CCS_Canu assembly (without Bionano scaffolding). Supports Figure 3.

**Supplemental Table S1.** Accession codes for assemblies and long-read data.

**Supplemental Table S2.** Accession codes for BACs.

**Supplemental Table S3.** Number of sequence variants (SNP, indels) between BAC-based sequence R gene loci and genome assemblies.

**Supplemental Table S4.** Gene annotation statistics.

## Supplementary Material

koab077_Supplementary_Data

## Notes

M.M., N.S., and J.S. designed the research. J.G., L.B.B., C.J.P., A.G.S., and J.E. performed the genome sequencing. M.M., J.J., C.P., and C.S.K. did genome assembly. T.L., T.W., H.G., K.F.X.M., and M.S. annotated gene and TEs. Z.T. and H.Š. performed the Bionano optical mapping. S.H., I.H.-P., and M.J.M. analyzed the resistance gene loci. J.S., J.G., A.G.S. and C.J.P. contributed sequence data or assembly algorithms. U.S. supervised the data management and submission. M.M. wrote the article with input from all co-authors.

The author responsible for distribution of materials integral to the findings presented in this article in accordance with the policy described in the Instructions for Authors (https://academic.oup.com/plcell) is Martin Mascher (mascher@ipk-gatersleben.de).
