# Supplementary material for: Long-read sequence assembly: a technical evaluation in barley
Source: Plant Cell. 2021 Mar 12;33(6):1888–906. doi: 10.1093/plcell/koab077 (PMC8290290; doi:10.1093/plcell/koab077)
Supplement: koab077_Supplementary_Data [file koab077_supplementary_data.zip › tpc.00883.2020-s02.pdf]

Long-read sequence assembly: a technical evaluation in barley

Martin Mascher, Thomas Wicker, Jerry Jenkins, Christopher Plott, Thomas Lux, Chu Shin Koh, Jennifer Ens, Heidrun Gundlach, Lori B. Boston, Zuzana Tulpová, Samuel Holden, Inmaculada Hernández-Pinzón, Uwe Scholz, Klaus F.X. Mayer, Manuel Spannagl, Curtis J. Pozniak, Andrew G. Sharpe, Hana Šimková, Matthew J. Moscou, Jane Grimwood, Jeremy Schmutz, Nils Stein

Corresponding authors: Martin Mascher (mascher@ipk-gatersleben.de) and Nils Stein (stein@ipk-gatersleben.de).

Review timeline:

**TPC2020-BR-00883**	Submission received:	Oct. 22, 2020
	1st Decision:	Nov. 24, 2020 *revision requested*
**TPC2020-BR-00883R1**	1st Revision received:	Jan. 13, 2021
	2nd Decision:	Jan. 27, 2021 *accept with minor revision*
**TPC2020-BR-00883R2**	2nd Revision received:	Jan. 27, 2021
	3rd Decision:	Jan. 27, 2021 *acceptance pending, sent to science editor*
	Final acceptance:	Feb. 28, 2021

REPORT: (The report shows the major requests for revision and author responses. Minor comments for revision and miscellaneous correspondence are not included. The original format may not be reflected in this compilation, but the reviewer comments and author responses are not edited, except to correct minor typographical or spelling errors that could be a source of ambiguity.)

TPC2020-BR-00883 1st Editorial decision – *revision requested* **Nov. 24, 2020**

We have received reviews of your manuscript entitled "Long-read sequence assembly for plant pan-genomics: a technical evaluation in barley." Thank you for submitting your best work to The Plant Cell. The editorial board agrees that the work you describe is substantive, falls within the scope of the journal, and may become acceptable for publication, pending revision and potential re-review.

We ask you to pay attention to the following points in preparing your revision:

Generally, the reviewers had only modest suggestions to improve the manuscript, and these would require careful editing and rewriting, perhaps with some re-analysis, but without major new experimental work. Please address all of the minor points in your Response to Reviewers document.

The major concern of the reviewers was essentially how the paper is framed. Based on the title and abstract, the reviewers indicate that they thought this paper would provide insight into pan-genomic studies of barley, but this is only briefly touched on in the paper. They also indicated that generating 'platinum' quality reference genomes may not provide a useful guideline for conducting pan-genome studies, as this would be far too expensive, at this point. Certainly, we all agreed in the post-review discussion that *de novo* assemblies with long reads is going to be the standard for future efforts for pan genome reconstruction. The most relevant functional implications of structural variation may reside in variants that do not affect coding portions of genes but rather their regulatory elements, many of which will reside in repetitive regions that can only be accessed through long read sequencing.

One of the concerns about how you've presented the work is that the 'recipe' you describe (~60x HiFi + BioNano + HiC) is not currently feasible (mainly for current cost considerations) for building a pan-genome across many diverse individuals, and it may be overkill. In the down-sampling analyses that you provided, ~20x HiFi data produced assemblies with similar N50 metrics (presumably capturing all of the gene space), and only 5x was sufficient for capturing ~80% of the genes. We discussed that it would be good for you to highlight this analysis and clarify that 'reasonable' amounts of long read data (~20x coverage) would be sufficient for pan-genome analyses in barley to capture all the new genes and repeats.

-- Reviewer comments:

[Reviewer comments shown below along with author responses]

TPC2020-BR-00883R1 1st Revision received Jan. 13, 2021

Reviewer comments and **author responses**:

Editor's comments:

The major concern of the reviewers was essentially how the paper is framed. Based on the title and abstract, the reviewers indicate that they thought this paper would provide insight into pan-genomic studies of barley, but this is only briefly touched on in the paper. They also indicated that generating 'platinum' quality reference genomes may not provide a useful guideline for conducting pan-genome studies, as this would be far too expensive, at this point. Certainly, we all agreed in the post-review discussion that *de novo* assemblies with long reads is going to be the standard for future efforts for pan genome reconstruction. The most relevant functional implications of structural variation may reside in variants that do not affect coding portions of genes but rather their regulatory elements, many of which will reside in repetitive regions that can only be accessed through long read sequencing.

We have changed the title and omitted the first sentence of the abstract to avoid raising expectations about a pan-genome paper.

One of the concerns about how you've presented the work is that the 'recipe' you describe (~60x HiFi + BioNano + HiC) is not currently feasible (mainly for current cost considerations) for building a pan-genome across many diverse individuals, and it may be overkill. In the down-sampling analyses that you provided, ~20x HiFi data produced assemblies with similar N50 metrics (presumably capturing all of the gene space), and only 5x was sufficient for capturing ~80% of the genes. We discussed that it would be good for you to highlight this analysis and clarify that 'reasonable' amounts of long read data (~20x coverage) would be sufficient for pan-genome analyses in barley to capture all the new genes and repeats.

We have clarified that we recommend to obtain ~20 HiFi coverage + Hi-C for assembling the genome of multiple individuals (ll. 480-482) of the revised manuscript). Bionano is an excellent tool for technical validation, but not strictly necessary for obtaining high-quality genome sequences as the contiguity of HiFi assemblies allows pseudomolecules only with Hi-C (see new Supplementary Figure 4). Note also that cost for the HiFi+Hi-C approach is lower than for the short-read TRITEX method that was used to assemble the 20 barley genome sequences recently reported by Jayakodi et al. (Nature 2020, doi: 10.1038/s41586-020-2947-8).

NOTE: all line numbers refer to manuscript with changes highlighted.

Reviewer #1:

Mascher report an improved, chromosome scale genome assembly for barley. The authors tested different long read sequencing technologies and genome assembly algorithms and found that circular consensus sequencing (or HiFi) reads produced the highest quality assembly at a lower cost and lowest computational requirements. HiFi reads were able to accurately assembly complex tandemly duplicated genes and long repeats, which is an important consideration for assembling a true pan-genome. These resources will be valuable to the barley and broader cereal research communities. The paper is technically robust and generally well-written, but I have a few concerns/suggestions that I feel should be addressed.

1. The authors structure this paper around generating pan-genomic resources for barley, but no pan-genome resources were generated, and the assembly comparisons are not necessarily useful for pan-genomics projects. I understand the link between optimizing technologies and algorithms for pan genome work, but I don't think the proper tests were carried out here to guide future pan-genome work. Instead, the authors provide a guide for assembling a 'platinum' quality chromosome-scale assembly. These guidelines could be applied to assembling a pan-genome for a species, but at present, most researchers use high coverage Illumina data or low coverage long read data to identify

accessory regions of the genome across the genetic diversity of a species. Producing dozens of 'platinum' quality, chromosome-scale assemblies for dozens or hundreds of diverse accessions is beyond the budget of most pan-genome projects (but this will likely become the standard in the future).

We have clarified that we recommend to obtain ~20 HiFi coverage + Hi-C for assembling the genome of multiple individuals (ll. 480-482) of the revised manuscript). Bionano is an excellent tool for technical validation, but not strictly necessary for obtaining high-quality genome sequences as the contiguity of HiFi assemblies allows pseudomolecules only with Hi-C (see new Supplementary Figure 4). Note also that cost for the HiFi+Hi-C approach is lower than for the short-read TRITEX method that was used to assemble the 20 barley genome sequences recently reported by Jayakodi et al. (Nature 2020, doi: 10.1038/s41586-020-2947-8).

2. A technical evaluation could be completed by running comparisons of sequencing coverage vs gene space assembly or better yet, generating data for several diverse barley accessions and running comparisons of coverage depth or data type to optimize identification of accessory regions compared to the reference. The latter would be a true technical evaluation of using long-read sequences to assemble a pan genome. Without either of these new analyses, I would suggest the authors restructure the paper with less of an emphasis around pan-genomics. I think this paper stands on its own merits, but I was disappointed after reading the paper based on my expectations from the title and abstract.

We have changed the title and omitted the first sentence of the abstract to avoid raising expectations about a pan-genome paper.

3. The CCS assembly was not polished using short read data as the low error rate of these consensus reads overcomes the need for polishing. The accuracy of the CCS assembly was supported by several lines of evidence, but I am curious if polishing using the Illumina data would catch any remaining errors or if it would introduce more errors into the assembly because of mis-alignments of the short Illumina data. Was this tested? I don't think this has been reported anywhere and barley may be a good system to test this. I think this could be a useful but non-essential addition to the manuscript.

In ll. 225-231 of the revised manuscript, we report the results of polishing the CCS_Canu assembly with Illumina data, which didn't improve accuracy. We have added reference to recent papers on human genome assembly that argue against polishing CCS assemblies with short-read data for the reasons stated by the referee (alignment issues).

4. Assembly quality in complex genome regions was assessed by aligning the different assemblies to Sanger-based BAC sequences. The assemblies are ranked for completeness in each region, but more detailed statistics are not presented. What was the average nucleotide identity compared to the sequenced BACs? If the genome assemblies are as complete/accurate as the authors claim, I would guess the BACs should match close to 100% (unless I am missing something here).

We have added Supplementary Figure 4 to report the number of SNPs and indels between the published BAC sequences and the contigs of our WGS assemblies. The best WGS assembly (CCS_Canu) had 22 SNPs and indels relative to the 261 kb BAC sequence (Sanger) of the *Mla* locus, i.e. less than one mismatch per Mb. Some loci were sequenced on the PacBio RS II (uncorrected long-reads) and may not be a gold standard for nucleotide-level accuracy (e.g. 1600 mismatches at *rps2*) or contained errors in the BAC assemblies (rpg4/Rpg5).

5. It is unclear why the ONT-based contigs were used to fill gaps in the CCS assembly. The CCS assembly had a higher contiguity and overall size, what is the rational for filling gaps with the ONT assembly? I would think this could introduce errors into the final assembly.

Our rationale was that ONT contigs may span some gaps in the CCS+Bionano scaffolds due to longer read lengths. We note the CCS assembly had no gaps, but 3.37 Mb of gap sequence (Table 5) was introduced by scaffolding with the Bionano map. Some of the gaps were small (< 50 kb), so they could be spanned by ONT contigs. To avoid introducing errors, we required that the gap size estimated by the Bionano map and the length of the filled-in sequence from the ONT assembly are very close. Even though ONT assembly may not be as accurate as the CCS assembly, the filled-in sequence is likely to provide to some useful information as opposed to N sequence.

6. Line 390-409. These down sampling analyses are probably the most relevant to pan-genomic studies as readers will want to know the 'minimum' coverage that is needed to assemble most of the gene space. If the authors proceed with framing this manuscript as a guide for pan-genome studies, these results should be highlighted in the abstract and perhaps additional QC metrics could be performed on the down sampled datasets. Remarkably, ~20x coverage of HiFi data produced assemblies with similar N50s as the full datasets, and only 4-5x coverage was able to resolve ~80% of high confidence genes. This was news to me, and I think many readers will also find these statistics useful.

We mention the key findings of our downsampling analysis in the abstract of the revised manuscript.

Minor:
Line 292. How many of the 35,827 high confidence genes are new in V3 compared to V2?

The coding sequences of 35,260 (98.4 %) of Morex V3 high-confidence gene models had near-complete alignments (>= 95 % alignment coverage, >= 99 % identity) to the V2 pseudomolecules, confirming that gene space presentation is very good also in short-read assemblies. We added these results to the revised manuscript (ll. 329-332).

Reviewer #2:

The paper is nicely written and contains interesting data that show the usefulness of new long read sequencing technologies for the assembly of large genomes. My only major revision is the following:

I would reinforce the argument that long read assemblies are much better because they give us uninterrupted scaffolds that coincide with contigs and give us access to the sequence content of repetitive regions. While in the past these regions were viewed as uninteresting because they contained what was then defined as "junk DNA", today we know that that DNA is often functionally relevant because it can play fundamental roles in regulatory circuits and that is a major source of genetic variation in plant genomes as a consequence of polymorphic transposable element insertions.

We completely agree with the reviewer on this point. Long-read sequence assemblies will underpin future studies on TE evolution and the roles of TEs in gene regulation.

All my minor revisions are embedded in the annotated PDF file.

We addressed the editorial changes in the PDF annotation.

We modified Figure 1 and Table 5 and added more explanation about assembling heterozygous genomes (ll. 526-529).

Reviewer #3:

Line 37 Sanger sequencing seems an odd technology to be referring to since it is not used in the large genome assemblies. Suggest deleting first sentence since defining the Pan genome is not particularly relevant to the paper. The relevant point for the paper is "Sequence assembly of large and repeat-rich plant genomes has been challenging ..." and reference to the pan genome is a distraction

We have removed the first sentence of the abstract and modified the title to avoid raising the expectation that this a pan-genome paper (i.e. reporting assembly of multiple accessions), rather than a technical paper whose results can instruct the design of future pan-genome studies.

Line 55 Given the authors on the paper the focus on TEs is not surprising but is getting pretty tedious as it continues to be a focus in cereal genomics. This paper ignores the biologically relevant repetitive loci of the rDNA and 5S rDNA which account for 80% of the RNA in all cells, and are part of the cell's most important engine for producing all proteins. The success claimed by the authors rings a bit hollow without these gene arrays being considered. The papers by Handa et al in wheat provide something of a template that could well be developed to a high level with the current assembly of the barley reference genome.

We agree that Handa et al. 2018 Plant Journal is the most detailed study of rDNA locus organization in a Triticeae

genome. Handa et al. found that the rDNA clusters are not well represented in the current wheat reference genome (IWGSC 2018), which is based on short-reads. Even the long reads used in the present study did not resolve the barley rDNA loci, which are fragmented into small contigs which have high coverage, indicating that identical rDNA copies were collapsed. We added a brief description of the missing 5H and 6H rDNA loci in ll. 338-340 of the revised manuscript.

The current manuscript focusses on a technical evaluation of assembly methods and constructing an updated reference genome for barley. Analyzing ribosomal loci was not our original focus. Now, we are indeed working on a follow-up study to analyze the prospects of obtaining telomere-to-telomere assemblies in barley. This work focusses on centromeric, telomeric and ribosomal repeat clusters. It is already becoming clear that even the most powerful genome sequencing and mapping technologies such as PacBio HiFi and Bionano optical mapping cannot fully resolve the rDNA repeat arrays, which will thus remain an obstacle to end-to-end sequence assembly in barley.

Lines 126- 128 "focusing on whether long-read approaches can (i) underpin the construction of chromosome-scale sequence assemblies in barley and (ii) scale to pan-genomics, i.e. the assembly of 10 to 100 genotypes". The reference to the pan-genome is a distraction from the very valuable contribution to manuscript makes to the area of cereal genomics.

We have removed the second part of the sentence.

Lines 156-158 - specific examples. Example of the MLA locus is satisfying to see reported. As noted above the authors need to address the rDNA and 5SrDNA arrays. In addition, an important locus such as the *Ppd* would be an advantage to report on with respect to its repetitive gene content

***PPD-1* has indeed been reported to reside in a complex, difficult-to-assemble locus in the wheat B genome, see the recent papers of Würschum et al. (BMC Genetics 2019) and Shimizu et al. (DNA Research https://doi.org/10.1093/pcp/pcaa152). As *PPD-H1* is an important gene for barley crop evolution, its allelic variation has been extensively studied, but to our knowledge copy-number or presence-absence variation has not been reported for this gene. The complete *PPD-H1* sequence was present as a single copy sequence in both the Morex V2 and V3 assemblies.**

Lines 168-174 Note also 213-240: "To compare base-level sequence accuracy between assemblies, we aligned high-confidence models of the current Morex V2 annotation to each assembly and required alignment threshold (99 % alignment / 100 % sequence identity). Our choice of gene model alignments as a benchmark of base-level accuracy was motivated by the observation that differences in gene space representation as approximated by alignment rates of full-length cDNAs and Isoseq reads (at 90 % coverage and 97 % identity) were minor, indicating that all assemblies captured the vast majority of genes." I again draw attention to the absence of the rDNA and 5SrDNA loci in the paper.

We have made our statement more precise: "… that all assemblies captured the vast majority of *protein-coding* genes."

Line 283 Fig 2 indicates HiC pseudomolecules but there is no mention in the text of HiC (one mention line 299) or methods

We have added a reference to the source of our Hi-C data (Mascher et al. 2017) and to the ENA accession number of the Hi-C reads in ll. 698-699.

Line 303 mention is made of the centromere repeats but nothing about the repetitive rDNA and 5SrDNA loci (sorry to be repeating myself)

We added a brief description of the missing 5H and 6H rDNA loci in ll. 338-340 of the revised manuscript.

Line 420 Terminology such as "out-perform", "best assembly" and "more accurate" are not appropriate in the area of large genome assemblies since these claims are routinely made on every new version of an assembly with the full knowledge that the claim is spurious. I would urge authors to report against the facts of the comparisons between the current and previous assemblies and let these facts speak for themselves.

We agree with reviewers that these controversial terms should be employed with great care. We checked each

occurrence:

Outperform (l. 258): We assert that the CCS is better than other assemblies we analyzed. The assemblies and criteria used in this assertion are defined in the first paragraph of the results section and it is only in this narrow and precise sense that we use the term "outperform".

More accurate (l. 411): We modified this sentence to read "… translate into pseudomolecules that are concordant with high-resolution genetic maps and correct errors of prior assembly versions".

Best assembly strategy (l. 327). We have modified this sentence that CCS is *currently* the best strategy, thus acknowledging that a better approach may become available in the future. We are confident that the present manuscript provides sufficient evidence that CCS delivers the best assemblies in barley right now.

Line 455 Please quote IWGSC 2018 as the correct reference for the wheat genome with respect to deploying the HiC technology.

We have added the IWGSC 2018 reference. The IWGSC 2014 citation indeed refers only to the applicability of chromosomal genomics to wheat.

TPC2020-BR-00883R1 2nd Editorial decision – *accept with minor revision* Jan. 27, 2021

We have received reviews of your manuscript entitled "Long-read sequence assembly: a technical evaluation in barley." On the basis of the advice received, the board of reviewing editors would like to accept your manuscript for publication in The Plant Cell. This acceptance is contingent on revision to address just the two minor points of Reviewer #2.

TPC2020-BR-00883R2 2nd Revision received Jan. 27, 2021

Reviewer comments and **author responses**:

Reviewer #2:

I am OK with the revisions made and I think the paper is fine now. I have just two minor comments:

1) Even though the focus on pan genome reconstruction was largely removed, I would still like to see a sentence in the abstract that points to the opportunities offered by the assembly strategy outlined in the paper to produce accurate and complete assemblies in multiple genomes of a species, thus paving the way to accurate pan genome reconstructions

We have changed the last sentence of the abstract to: "Long-read assembly can underpin the construction of accurate and complete sequences of multiple genomes of a species to build pan- genome infrastructures in Triticeae crops and their wild relatives."

2) LINE 311-312 modify as follows:

The coding sequences of 35,260 (98.4 %) Morex V3 high-confidence gene models had near- complete alignments....

We have removed "of" before "Morex V3".

TPC2020-BR-00883R2 3rd Editorial decision – *acceptance pending* Jan. 27, 2021

We are pleased to inform you that your paper entitled "Long-read sequence assembly: a technical evaluation in barley" has been accepted for publication in The Plant Cell, pending a final minor editorial review by journal staff.

Final acceptance from Science Editor Feb. 28, 2021
