# Supplementary material for: Long-read sequence assembly: a technical evaluation in barley
Source: Plant Cell. 2021 Mar 12;33(6):1888–906. doi: 10.1093/plcell/koab077 (PMC8290290; doi:10.1093/plcell/koab077)
Supplement: koab077_Supplementary_Data [file koab077_supplementary_data.zip › tpc.00883.2020-s03.pdf]

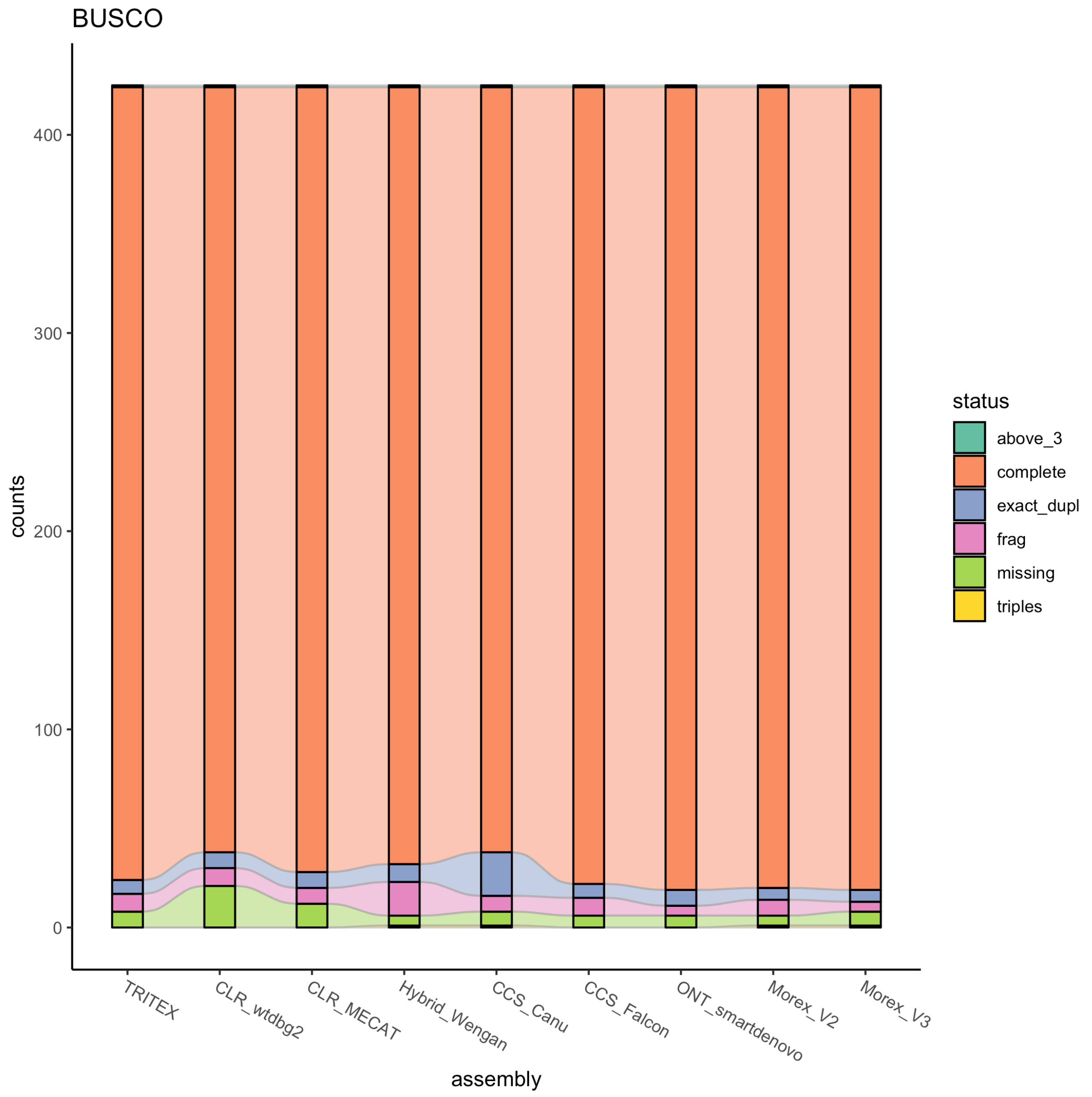

Supplemental Figure S1: BUSCO assessment of different sequence assemblies.

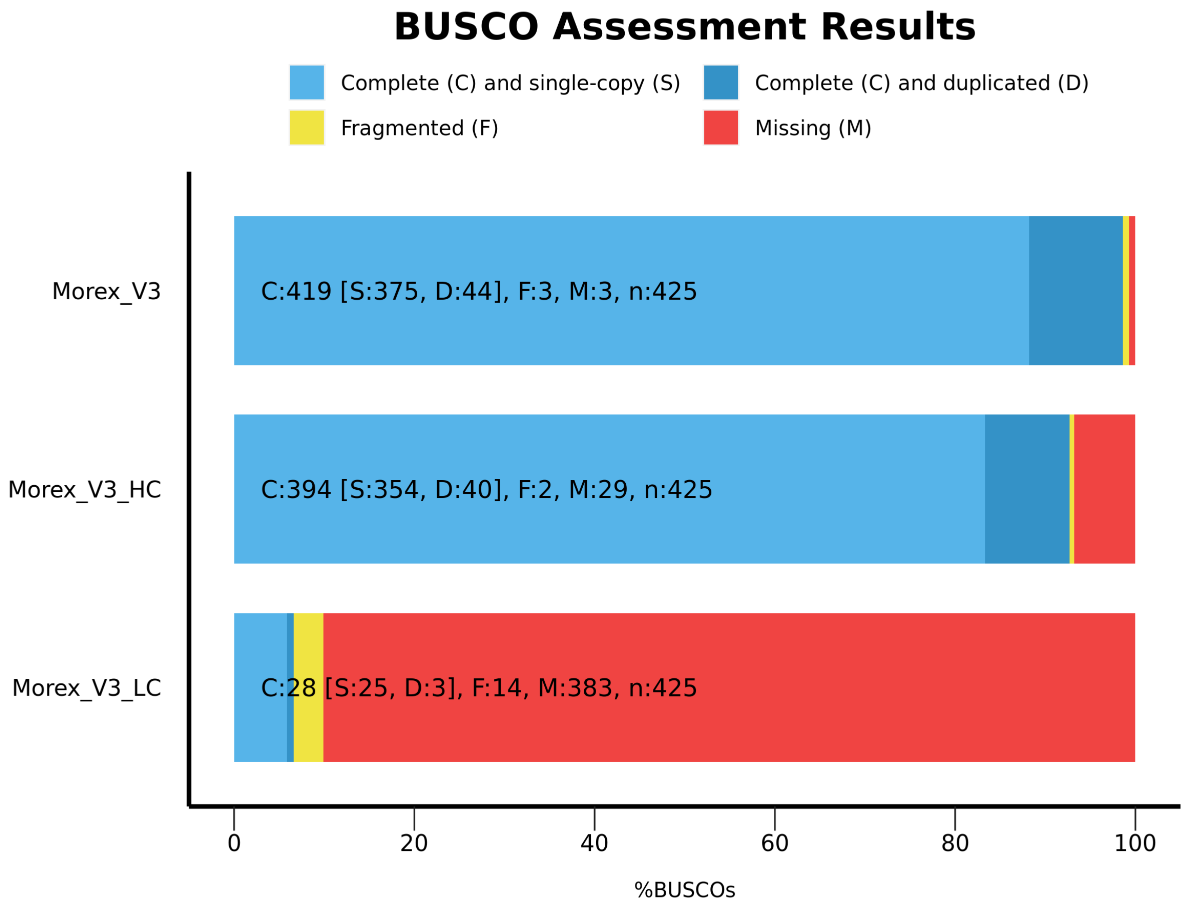

Supplemental Figure S2: BUSCO assessment of the structural gene annotation of the Morex V3 pseudomolecules. Note that multiple isoforms were annotated for some genes so that a higher proportion of duplicates is found. HC: high-confidence; LC: low-confidence.

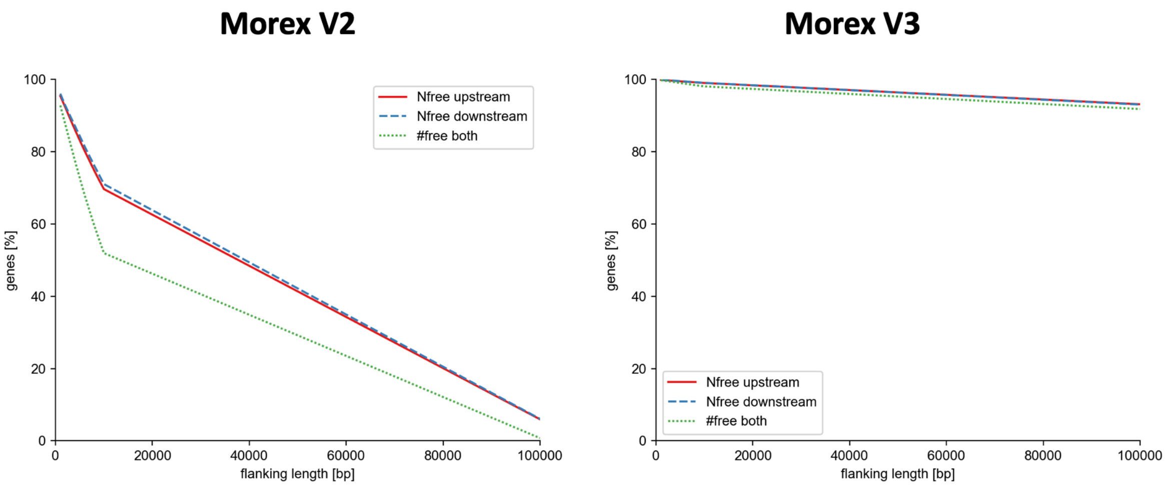

Supplemental Figure S3: Gap-free sequence in upstream and downstream regions of genes in Morex V2 and Morex V3.

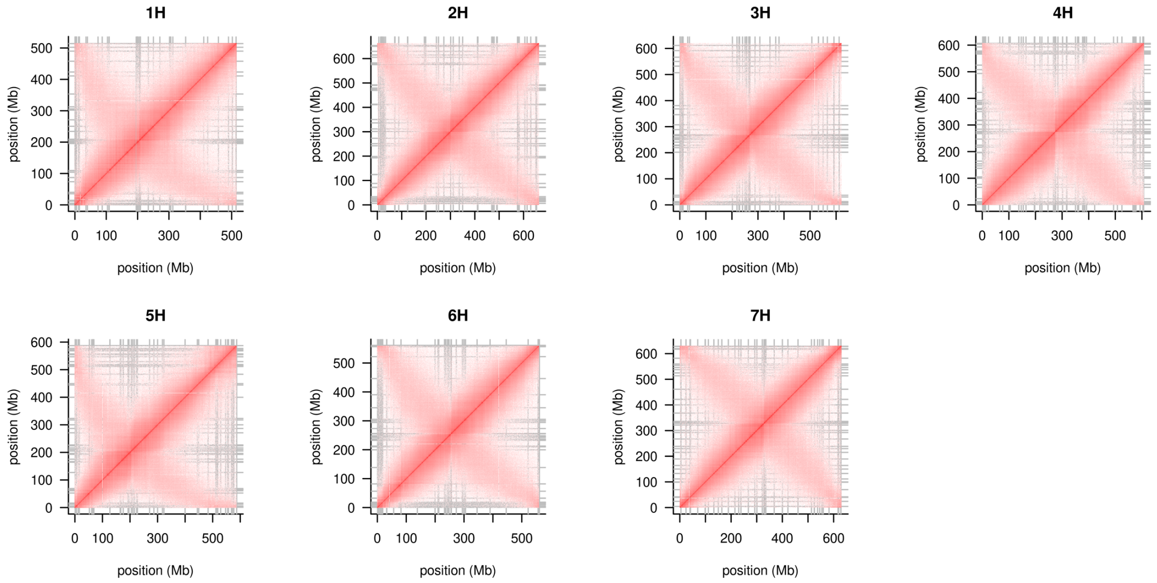

Supplemental Figure S4: Hi-C contact matrices for pseudomolecules constructed from the CCS_Canu assembly (without Bionano scaffolding). Grey lines indicate contig boundaries. The absence of off-(anti)-diagonal signals supports the accuracy of the assembly.

Supplemental Table S1: Accession codes for assemblies and long-read data.

Study	Assembly	Sample	Experiment/Contigs	Run/Chromosome	Type	Name
PRJEB40580	GCA_904848305	ERS5134606	CAJHBX010000001-CAJHBX010001207	-	Genome	CCS_Falcon Morex
PRJEB40581	GCA_904848295	ERS5134607	CAJHCA010000001-CAJHCA010007796	-	Genome	CCS_Canu Morex
PRJEB40582	GCA_904848315	ERS5134608	CAJHBZ010000001-CAJHBZ010014177	-	Genome	CLR_wtdbg2 Morex
PRJEB40583	GCA_904848275	ERS5134609	CAJHCB010000001-CAJHCB010001594	-	Genome	CLR_mecat Morex
PRJEB40584	GCA_904848285	ERS5134610	CAJHBY010000001-CAJHBY010000895	-	Genome	ONT_smartdenovo Morex
PRJEB40585		ERS5134611		-	Genome	Hybrid_Wengan Morex
PRJEB40586	GCA_904848325	ERS5134612	CAJHCC010000001-CAJHCC010494276	-	Genome	TRITEX Morex
PRJEB40587	-	ERS5135541-ERS5135545	ERX4582061-ERX4582065	ERR4659245-ERR4659249	WGS	Morex V3 CCS raw reads
PRJEB40588	-	ERS5135519-ERS5135540	ERX4582039- ERX4582060	ERR4659223-ERR4659244	WGS	Morex V3 ONT raw reads
PRJEB40589		ERS5142877			Genome	Morex_V3_assembly pseudomolecules

Supplemental Table S2: Accession codes for BACs.

Locus	BACs	NCBI identifier	Sequencing strategy	Coverage
Mla	HVVMRXALLHA0711N16 (MoKI0190E06)	AF427791	Subcloning, Sanger sequencing	n.a.
Mla	HVVMRXALLHA0080H14 (MoKI0020M19)	AF427791	Subcloning, Sanger sequencing	n.a.
Rpg1	HVVMRX83KHA0063I13 (MoHI0244M13)	AF509748	Subcloning, Sanger sequencing	n.a.
Rpg1	HVVMRX83KHA0161H16 (MoHI0607P19)	AF509748	Subcloning, Sanger sequencing	n.a.
rpg4/Rpg5	HVVMRXALLMA0543P19 (MoKI0144P03)	EU812563	Subcloning, Sanger sequencing	n.a.
rpg4/Rpg5	HVVMRXALLMA0116G10 (MoKI0029A11)	EU812563	Subcloning, Sanger sequencing	n.a.
rps2	HVVMRX83KHA0091B04 (MoHI0347G17)	PRJNA664952	PacBio RS II	4236
rps2	HVVMRX83KHA0179H16 (MoHI0671F17)	PRJNA664952	PacBio RS II	2424
rps2	HVVMRX83KHA0215K06 (MoHI0802F14)	PRJNA664952	PacBio RS II	2471
rps2	HVVMRX83KHA0217M01 (MoHI0808K20)	PRJNA664952	PacBio RS II	2774
Rps6	HVVMRXALLMA0379I20 (MNMA0379I20)	PRJNA664952	PacBio RS II	1543
Rps6	HVVMRXALLMA0135D15 (MNMA0135D15)	PRJNA664952	PacBio RS II	929
Rps6	HVVMRXALLMA0276D15 (MNMA0276D15)	PRJNA664952	PacBio RS II	858
Rps8	HVVMRX83KHA0047E04 (MOKI0047E04)	PRJNA664952	PacBio RS II	~1400
Rps8	HVVMRX83KHA0046I18 (MOKI0046I18)	PRJNA664952	PacBio RS II	~1000
Rps8	HVVMRX83KHA0188J02 (MOKI0188J02)	PRJNA664952	PacBio RS II	~550

Supplemental Table S3: Number of sequence variants (SNP, indels) between BAC-based sequence *R* gene loci and genome assemblies.

Assembly	*Mla*	*Rpg1*	*rpg4/Rpg5*	*rps2*	*Rps6*	*Rps8-1*	*Rps8-2*
CCS_Canu	22	0	7907	1600	972	16	1
CLR_MECAT	26	0	7904	1932	F	18	1
TRITEX	F	0	7913	33592	F	5833	F
Hybrid_Wengan	F	0	F	F	F	644	F
CLR_wtdbg2	F	0	7908	F	2382	25	19
ONT_smartdenovo	2416	0	8699	6211	3888	580	512
CCS_FALCON	F	0	19664	1599	972	5833	2
F: Fragmented sequence relative to BAC contig							
Note: The published *rpg4/Rpg5* BAC assembly has an error in the assembly							

Supplemental Table S4: Gene annotation statistics.

	Complete			High-confidence (HC)			Low-confidence (LC)	
	Total	Average		Total	Average		Total	Average
Number of genes	81687	NA		35827	NA		45860	NA
Number of monoexonic genes	46175	NA		11537	NA		34638	NA
Transcripts per gene	83990	1.03		37963	1.06		46027	1
Coding transcripts per gene	83990	1.03		37963	1.06		46027	1
CDNA lengths	81400273	969.17		53654543	1,413.34		27745730	602.81
CDNA lengths (mRNAs)	81400273	969.17		53654543	1,413.34		27745730	602.81
CDS lengths	71837620	855.31		45079806	1,187.47		26757814	581.35
CDS lengths (mRNAs)	NA	855.31		NA	1,187.47		NA	581.35
CDS/cDNA ratio	NA	92.96		NA	86.24		NA	98.5
Monoexonic transcripts	46175	634.6		11537	963.96		34638	524.9
MonoCDS transcripts	47011	618.84		12246	905.33		34765	517.93
Exons per transcript	240677	2.87		169194	4.46		71483	1.55
Exons per transcript (mRNAs)	3164	2.87		3027	4.46		2010	1.55
Exon lengths	NA	338.21		NA	317.12		NA	388.14
Exon lengths (mRNAs)	NA	338.21		NA	317.12		NA	388.14
Intron lengths	NA	564.07		NA	577.67		NA	493.98
Intron lengths (mRNAs)	NA	564.07		NA	577.67		NA	493.98
CDS exons per transcript	2775	2.8		2648	4.32		1781	1.54
CDS exons per transcript (mRNAs)	2775	2.8		2648	4.32		1781	1.54
CDS exon lengths	71837620	305.88		45079806	274.8		26757814	377.88
CDS Intron lengths	83014426	550.25		70971472	562.89		12042954	485.92
5'UTR exon number	83990	0.26		37963	0.54		46027	0.04
3'UTR exon number	83990	0.26		37963	0.53		46027	0.04
5'UTR length	3166948	37.71		2816553	74.19		350395	7.61
3'UTR length	6395705	76.15		5758184	151.68		637521	13.85
Stop distance from junction	NA	3.6		NA	5.13		NA	2.35
Intergenic distances	NA	49,823.15		NA	114,705.99		NA	91,228.58
Intergenic distances (coding)	NA	49,823.15		NA	114,705.99		NA	91,228.58
